# Trust and COVID-19 vaccine hesitancy

**DOI:** 10.1038/s41598-023-35974-z

**Published:** 2023-06-07

**Authors:** Vincenzo Carrieri, Sophie Guthmuller, Ansgar Wübker

**Affiliations:** 1 Department of Political and Social Sciences, University of Calabria, Arcavacata di Rende (CS), Italy; 2 RWI-Leibniz Institute for Economic Research, Essen, Germany; 3 IZA, Bonn, Germany; 4 Vienna University of Economics and Business, Vienna, Austria; 5 Hochschule Harz, Wernigerode, Germany

**Keywords:** Health policy, Preventive medicine, Health care economics

## Abstract

This article uses novel data collected on a weekly basis covering more than 35,000 individuals in the EU to analyze the relationship between trust in various dimensions and COVID-19 vaccine hesitancy. We found that trust in science is negatively correlated, while trust in social media and the use of social media as the main source of information are positively associated with vaccine hesitancy. High trust in social media is found among adults aged 65+, financially distressed and unemployed individuals, and hesitancy is largely explained by conspiracy beliefs among them. Finally, we found that the temporary suspension of the AstraZeneca vaccine in March 2021 significantly increased vaccine hesitancy and especially among people with low trust in science, living in rural areas, females, and financially distressed. Our findings suggest that trust is a key determinant of vaccine hesitancy and that pro-vaccine campaigns could be successfully targeted toward groups at high risk of hesitancy.

## Introduction

High levels of vaccination coverage in populations are necessary to end the coronavirus emergency phase. Indeed, evidence has accumulated that available vaccines for COVID-19 are effective to reduce infection rates and—even more important—to prevent severe illness and death^1^. However, while in many countries in the world vaccines are still not yet available for everyone, a non-negligible share of individuals is hesitant to undertake vaccination for COVID-19 in most developed countries. Just before its entry into the global market, a nationwide survey reported acceptance rates for COVID-19 vaccines ranging from 55 to 90%^2^. Actual vaccination rates are almost in line with those expectations and current vaccination rates range from 30 to 95% in Europe with an average of 75% of persons vaccinated as of September 2022^3^.

Unfortunately, vaccine hesitancy was already a growing concern before the COVID-19 pandemic. In 2018, the World Health Organization included it among the top ten threats to public health globally^4^. Vaccine hesitancy still represents a threat also to the eradication of re-emergent vaccine-preventable diseases like measles and rubella^5,6^. Previous research has found that several individual-level factors are strongly associated with vaccine hesitancy, such as low income and poor education, certain political or religious orientations, and perceived risk in terms of efficacy and safety^7–11^.

However, in the case of the COVID-19 vaccine, the reasons for hesitancy remain even more complex^12^. On the one hand, the persistence of a pandemic period that has caused major restrictions on freedom of movement and socialization for many individuals should incentivize individuals to vaccinate. On the other hand, the approval of a new vaccine very quickly could worry the most undecided individuals and prompt them not to vaccinate. Available evidence suggests indeed that hesitancy might be explained by a lack of COVID-19 vaccine confidence and complacency as well as sociodemographic and psychological determinants^13^. This was found especially during the period in which vaccines were approved under an Emergency Use Authorization^14^. These findings suggest that a relevant factor explaining COVID-19 vaccine hesitancy might be effectively represented by an inherent lack of trust.

In economics and social sciences, trust is generally defined as the expectation that others will behave cooperatively and trustworthily. Trust is an important factor in economic transactions because it can reduce the costs of monitoring and enforcing contracts, and facilitate trade and exchange. Trust can have several dimensions, including interpersonal trust (the trust between individuals), institutional trust (the trust in the institutions such as the legal system or regulatory agencies), and social trust (the trust in the society (see e.g. Arrow (1992)^15^ for the role of trust in economic transactions or Putnam (1993)^16^ with regard to trust in the context of social capital.) In our paper, we rely on trust variables available in the data, therefore, we ultimately use a data-driven approach.

There is much anecdotal evidence that trust in its various dimension—e.g. into public institutions, science, media, social media—plays a major role in vaccine hesitancy. However, to the best of our knowledge, there is little evidence on the link between trust in various dimensions and COVID-19 vaccine hesitancy. Tan, Straughan, and Cheong (2022) used data from a nationally representative panel survey of Singaporeans aged 56–75 and found that trust in formal sources of information (e.g. government sources) predicts vaccination status among respondents. However, contrary to expectations, trust in social media and informal sources (family and friends), and perceived social support are found to predict vaccination status very badly^17^. Sturgis et al. (2021), using representative survey data covering 126 countries, showed instead a large positive macro-level association between COVID-19 vaccine confidence and both the average level of trust in science in a country and its variability around that average^18^. Betsch et al. (2008) assessed confidence in vaccination as a potential determinant of vaccination using the question "How much do you trust vaccination?" as a trust variable. They found that trust is strongly associated with vaccination confidence (belief in the safety and efficacy of vaccines) and collective responsibility (feeling collectively sensitive to getting vaccinated)^19^. Roozenbeek et al. (2020) examined the association between vulnerability to misinformation, vaccine hesitancy, and trust in scientists among individuals in different countries. They found that individuals who are more susceptible to misinformation about COVID-19 are more hesitant to be vaccinated^20^. Related, Freeman et al. (2020) examined the association between distrust of institutions, conspiracy theories related to coronavirus, and vaccination intentions related to COVID-19^21^. They found that belief in conspiracy theories and distrust of authorities can undermine efforts to promote public health during a pandemic and highlight the importance of disseminating accurate information and building trust in government and public health authorities.

Our paper contributes to the literature using novel European survey data including rich information on different trust dimensions (media, government, science, European Union, social media, etc.), COVID-19 immunization intentions along with reasons for hesitancy, as well as socioeconomic, demographic, and health information from EU countries covering more than 35,000 individuals interviewed during the first quarter of 2021, including information on the day of the interview. In a first step, we use multivariate regression methods to assess the factors associated with COVID-19 vaccination hesitancy focusing on the relationship between trust in various dimensions and vaccine hesitancy. In a second step, we look at the mechanisms of trust formation by investigating the role of demographic, socio-economic, health characteristics, and media use. Lastly, we study the effect of the temporary suspension in the AstraZeneca vaccine administration in March 2021 after concerns about potential side effects in the form of blood clots^22,23^.

There is an increasing but relatively scarce evidence on the effect of this suspension on vaccine hesitancy based on different research designs. Deiana et al. (2022) found that the suspension caused a substitution effect towards Pfizer-BioNTech (PB) vaccine in Italy. The substitution effect was more pronounced in regions displaying greater attention to vaccine side effects as detected through Google searches^24^. Using data on vaccine acceptance in eight Western countries obtained on a daily basis, Petersen et al.(2022) also found that these suspensions—and associated news—increased vaccine hesitancy in several countries, and part of this decrease happened in response to the ban introduced in other countries^25^. Agosti et al. (2022) compared vaccine hesitancy in countries that suspended AstraZeneca to countries that did not suspend AstraZeneca before and after the ban in a differences-in-differences setting. They found that the AstraZeneca controversy and its suspension, increased vaccine hesitancy but quite modestly^26^.

In this paper, in order to rule out spillover effects of the suspensions across countries and the local availability of alternative vaccines (i.e. Pfizer-BioNTech (PB)) that might be subject to supply effects, we take a different route. We then complement existing evidence by using an event study approach that compares intentions to vaccinate on a weekly basis from February to April 2021 before and after the week in which suspensions were concentrated in Europe (12–18 March 2021). This approach also allows us to transparently assess pre-trends and study the association of the suspension with vaccine hesitancy in a dynamic perspective. We study the potentially heterogeneous reactions to the suspension and the interplay between trust in science, vaccine hesitancy, and individual-level factors traditionally correlated with higher hesitancy such as gender, area of residence (rural vs. urban), economic status, and health status. To the best of our knowledge, no previous studies have looked at these effects on a large sample of European citizens, so far.

## Results

### Trust and individual-level factors associated with covid-19 vaccine hesitancy

**Figure 1:**
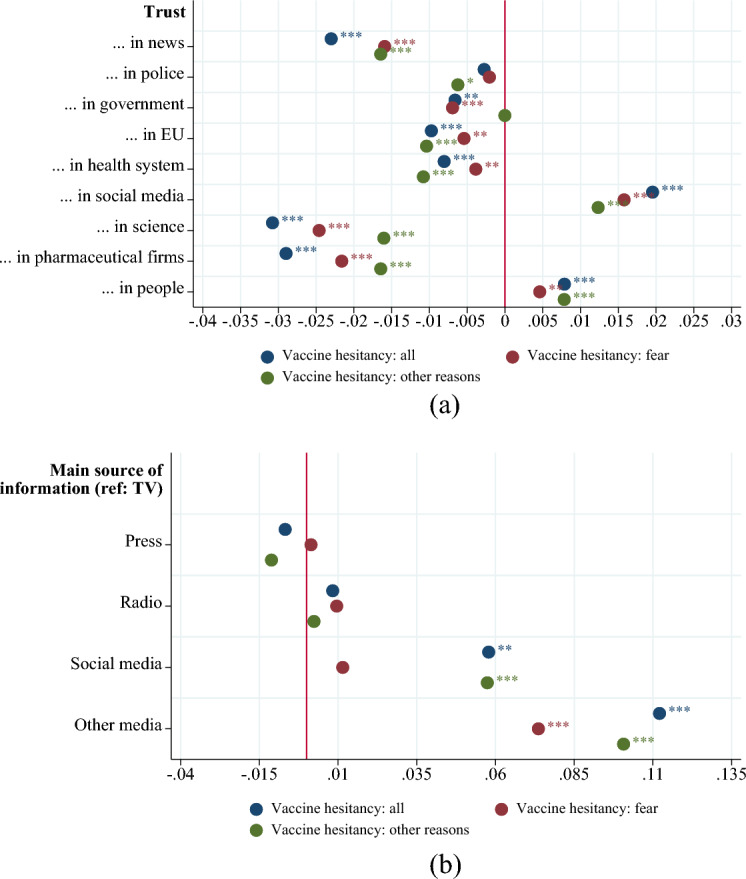
Correlates of COVID-19 vaccine hesitancy: Trust and main source of information. *Note*: The figure reports the coefficient estimates for the trust variables (graph a) and for the main source of information (graph b) of the likelihood to be vaccine hesitant (see Eq. (1) in section “Methods”). The blue dots show the coefficients for all reasons of vaccine hesitancy, the red dots show the coefficients for fear as main reason for vaccine hesitancy and the green dots show the coefficients for other reasons (mainly conspiracy reasons) for vaccine hesitancy. The full estimation results are available in Table A.1 in the Appendix. *p < 0.1, **p < 0.05, ***p < 0.01.

Figure 1 displays regression coefficients of the factors associated with COVID-19 vaccine hesitancy (unlikely/very unlikely to undertake COVID-19 vaccine) and the associated statistically significance levels. We found that trust in all dimensions is negatively correlated with vaccine hesitancy except for trust in people, and more importantly, trust in social media. For the latter, an increase of one point in the trust scale—spanning from 1 to 10—is found to significantly increase vaccine hesitancy by 2 percentage points (two-sided *p*-value = 0.000). On the contrary, trust in science is found to be the most relevant predictor of low vaccine hesitancy among all trust variables: an increase of 1 point in the trust scale is associated with a reduction of vaccine hesitancy by around 3 percentage points (two-sided *p*-value = 0.000). The average of the vaccine hesitancy variable is 0.255 (see Table A.7), i.e. 25.5% of individuals are vaccine-hesitant in our sample. A decrease in vaccine hesitancy of 3 percentage points suggests that if trust in science would increase by one standard deviation (2.46, see Table A.7), vaccine hesitancy would be reduced by 25%, i.e. the share of vaccine hesitancy would pass from 25.5 to 19% approximately. We stratify our sample by different reasons for not vaccinating related to trust. We divide between non-vaccinating reasons that relate to "fear" and "others—mainly conspiracy beliefs". “Fear” measures if a person answered “Yes” to the statements: “Reason for not taking vaccine: I am worried that it will make my health issues worse” and “Reason for not taking vaccine: I do not trust the safety of the vaccine”. “Others—mainly conspiracy beliefs” measures if a person answered “Yes” to the statements: “Reason for not taking vaccine: I think the risk of COVID-19 is exaggerated”; “Reason for not taking vaccine: I think COVID-19 doesn't exist” and “Reason for not taking vaccine: Other reason”. For both types of trust, social media and science, “fear of vaccine” as reason for hesitancy, is found to be more predictive than “conspiracy reasons”. We also stratify the sample according to the most relevant trust dimensions associated with hesitancy, i.e. high trust in social media only vs. high trust in science only. Results are presented in Table A.2 in the Appendix. We found that people with high trust in social media only are found to be more hesitant compared to people with high trust in both science and social media (two-sided *p*-value = 0.000). Interestingly, we also found that people with low trust at all are much more hesitant compared to people with high trust in both science and social media (two-sided *p*-value = 0.000).

Relatedly, the lower panel of Fig. 1 shows that people who use social media as main source of information are found to be much more hesitant compared to people who use classical media sources (TV, Press and Radio) and conspiracy reasons are predominant for this group (two-sided *p*-value = 0.008).

**Figure 2:**
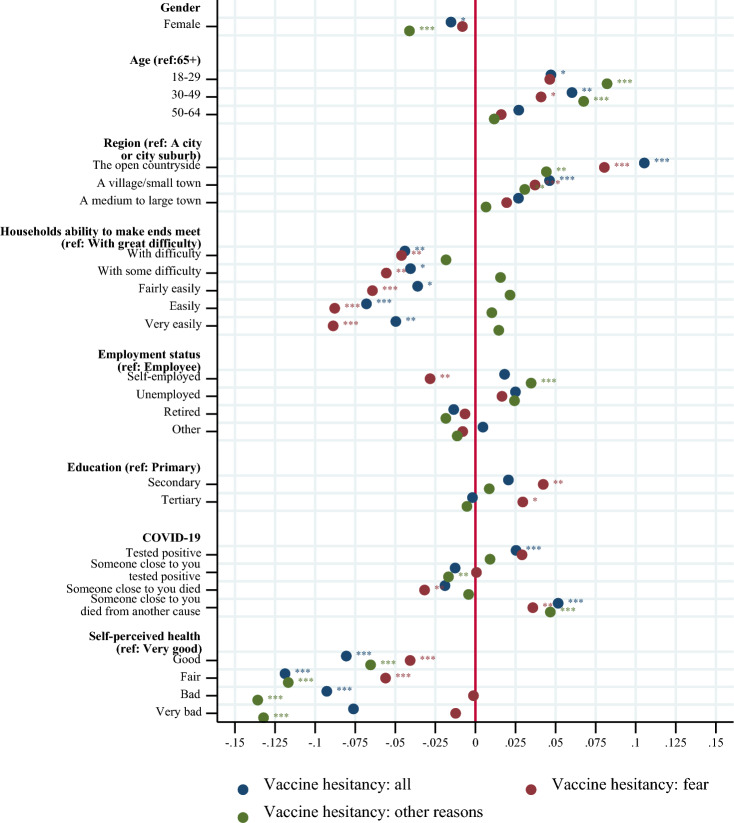
Correlates of COVID-19 vaccine hesitancy: health, demographic, and socioeconomic characteristics. *Note*: The figure reports the estimates for the set of demographic, socioeconomic and health-related variables of the likelihood to be vaccine hesitant (see Eq. (1) in section “Methods”). The blue dots show the coefficients for all reasons of vaccine hesitancy, the red dots show the coefficients for fear as main reason for vaccine hesitancy and the green dots show the coefficients for other reasons (mainly conspiracy reasons) for vaccine hesitancy. The full estimation results are available in Table A.1 in the Appendix. *p < 0.1, **p < 0.05, ***p < 0.01.

For what concerns the other correlates of vaccine hesitancy, Fig. 2 displays that males (two-sided *p*-value = 0.052), people below 50 (age group 18–29, two-sided *p*-value = 0.098, age group 30–49, two-sided *p*-value = 0.014), people who live in open-countryside (two-sided *p*-value = 0.000) and in a village or a small town (two-sided *p*-value = 0.000) are found to be more hesitant than their counterparts. Vaccine hesitancy and the level of financial difficulties are positively correlated, people with great financial difficulties are more hesitant compared to people with less financial difficulties (two-sided *p*-values < 0.01). We also found that self-employed are generally more hesitant, and significantly for conspiracy reasons (two-sided *p*-value = 0.008), while we did not detect a neat educational gradient in vaccine hesitancy. Finally, own experience with COVID-19, experience with COVID-19 among close persons and own health are associated with hesitancy but in a heterogeneous way. In particular, people with a previous diagnosis of COVID-19 are found to be significantly more hesitant to vaccinate (two-sided *p*-value = 0.000) presumably because they are partly immunized, people reporting someone close to them tested positive or died from COVID-19 are found to be less hesitant (but not statistically significant), and people reporting someone close to them died from another cause are found to be more hesitant (two-sided *p*-value = 0.000). The latter might be related to the skepticism about the codification of deaths from versus with COVID-19.

Results also vary with the reason for hesitancy (fear of vaccine versus other reasons, which mainly reflects the reason “conspiracy beliefs”). E.g., for people with bad or very bad health compared to the ones in very good health the differences are very pronounced regarding the different reasons for hesitancy. Table A.1 in the Appendix shows the coefficients and standard errors for all variables again in a condensed form. As a robustness check, we estimated the model with COVID-19 vaccine hesitancy as continuous and found that the results are qualitatively in line with those obtained by dichotomizing the dependent variable (results are available upon request).

### Factors associated with trust formation

**Figure 3:**
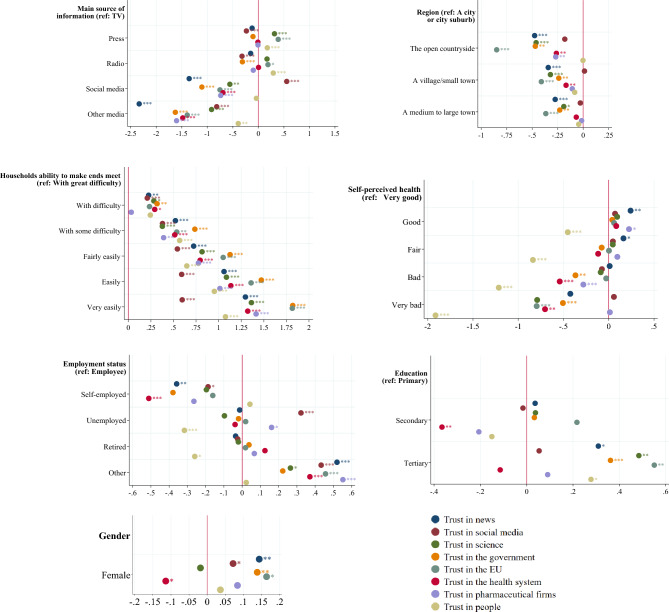
Correlates of trust: Role of information, demographic, health, and socioeconomic factors. *Note*: The figure reports the estimates for the main source of information use, the set of demographic, socioeconomic and health-related variables associated with the level of trust (in news, in social media, in science, in the government, in the EU, in the health system, in pharmaceutical firms, and in people, see Eq. (2) in section “Methods”. The full estimation results are available in Table A.3 in the Appendix. *p < 0.1, **p < 0.05, ***p < 0.01.

In Fig. 3, we plot regression coefficients of the main determinants of trust formation in various dimensions. First, we found that people using social media as main source of information have significantly less trust in the health system (two-sided *p*-value = 0.000, the EU (two-sided *p*-value = 0.002), the government (two sided *p*-value = 0.003), the news (two-sided *p*-value = 0.000), science (two-sided *p*-value = 0.010), and pharmaceutical firms (two sided *p*-value = 0.001) compared to traditional media users (reference group TV users) but—not surprising—more trust in social media (two-sided *p*-value = 0.000). The effects are quantitatively substantial and amount to between 0.5 and 1.5 points on a Likert scale of 1–10. We further found that: (i) people who live in the open countryside (compared to people in urban areas, two-sided *p*-values < 0.01), (ii) people with great financial difficulties to make ends meet (compared to people with fewer or hardly any problems in this regard, two-sided *p*-values < 0.01), (iii) people in poor or very poor health (compared to people in better health, two-sided *p*-values < 0.01), and (iv) less educated (compared to more educated people, two-sided *p*-values < 0.01) have generally significantly less trust in public institutions (e.g. science, the government, the health system or the EU) whereas unemployment status is significantly correlated with higher trust in social media (two-sided *p*-value = 0.002). A somewhat heterogeneous picture emerges for age groups, for women and for people who have had COVID-19 themselves or who have had family cases of deaths from COVID-19 or from other diseases (See Fig. A.1 and Table A.3 in the Appendix, for a tabular representation of coefficients and standard errors).

### AstraZeneca temporary suspension and vaccine hesitancy

**Figure 4:**
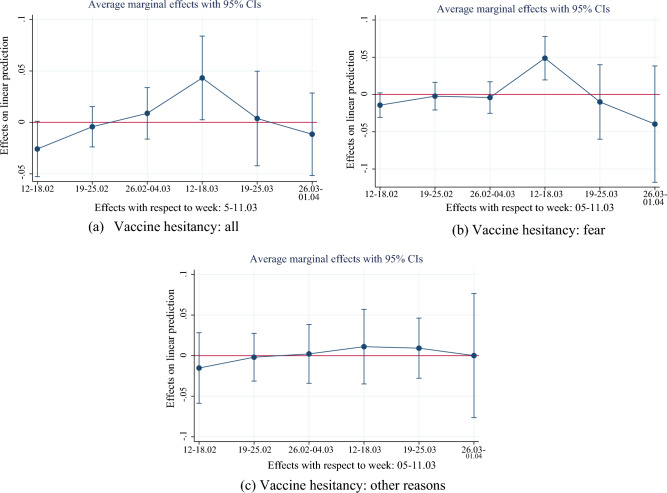
AstraZeneca ban and vaccine hesitancy. *Note*: The figure shows the average marginal effects (AME) for the impact of the week dummies based on an event study regression (see Eq. (1) in section “Methods”). The reference week is “5–11.03” that is the week immediately prior to the introduction of the AstraZeneca ban. The left graph (**a**) shows the AME for all reasons of vaccine hesitancy, the right graph (**b**) shows the AME for fear as main reason for vaccine hesitancy and the graph at the bottom (**c**) shows the AME for other reasons (mainly conspiracy reasons) for vaccine hesitancy. The vertical bars show the upper and lower bounds of the 95 percent confidence intervals of the estimation coefficients.

In Fig. 4, we show event-study coefficients of the AstraZeneca ban on vaccine hesitancy. We found that the temporary suspension of AstraZeneca was associated with a significant increase in hesitancy by 4 percentage points in the week just after the suspension (two-sided *p*-value = 0.038, Table A.4) while we did not find evidence of pre-existing significant trends. This result reassures us about the solidity of our research design. The effect seems to be mostly short-lived and highly concentrated in the week just after the suspension, on average. In Table A.5 in the Appendix, we report the event study analysis of the AstraZeneca suspension week interacted with the variable measuring trust in science. As shown in Table A.1, the trust variable was the one mostly associated with higher hesitancy; therefore, an interaction analysis helps us unravel the heterogeneous response to the ban among different levels of trust in science.

**Figure 5:**
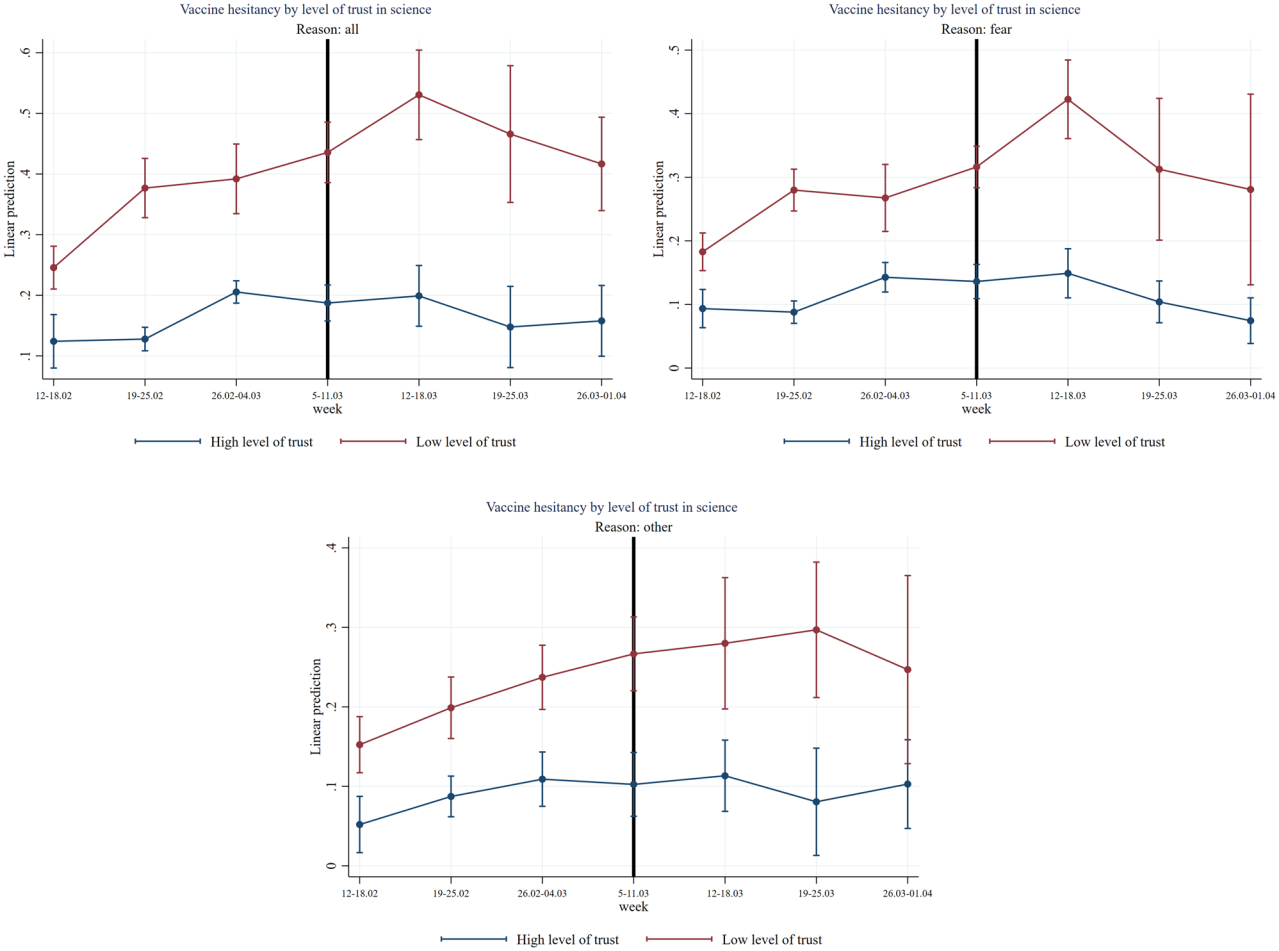
Effect of AstraZeneca ban by level of trust in science. *Note*: The figure shows estimation coefficients for the impact of the week dummies before and after the introduction of the AstraZeneca ban for weeks y = − 4 until y =  + 2 based on an event study regression according to Eq. (1) (see section “Methods”). The vertical line indicates the week prior to the week of the AstraZeneca ban. The left graph shows estimation coefficients for all reasons for vaccine hesitancy, the right graph shows estimation coefficients for fear as main reason for vaccine hesitancy and the graph at the bottom shows regression coefficients for other reasons (mainly conspiracy reasons) for vaccine hesitancy. The red line shows the results for the group with a low level of trust in science (having a score within the first or second country-specific tercile on a 1–10 Likert scale) and the blue line shows the results for the group with high level of trust (having a score within the third country-specific tercile on a 1–10 Likert scale). The vertical bars show the upper and lower bounds of the 95 percent confidence intervals of the estimation coefficients.

Figure 5 displays several interesting facts: First, people with low trust in science (having a score within the first or second country-specific tercile on a 1–10 Likert scale) compared to people with high trust in science (having a score within the third country-specific tercile on a 1–10 Likert scale) are found to have more than 20 percentage point lower intention to get vaccinated against Covid-19 (two-sided *p*-value = 0.000). Second, the suspension is found to be significantly associated with an increase of more than 8 percentage points in vaccine hesitancy in the week after the suspension in the group of low trust people compared to the group of high trust people (two-sided *p*-value = 0.027, see Table A.5). This effect is very large and—as shown by the flat blue line—the group of high trust people seems to be unaffected in their decision to non-vaccinate by the suspension of AstraZeneca. Third, fear of vaccine is found to be the most relevant reason for this jump in hesitancy, associated with a significant increase of more than 9 percentage points in vaccine hesitancy in the low trust group of people (two-sided *p*-value = 0.030, see Table A.5). Other reasons for vaccine hesitancy—mainly conspiracy beliefs—are found to be much less relevant for the jump in vaccine hesitancy for the low trust group of people as shown in the bottom chart of Fig. 5 and they are not statistically significant effects at conventional levels (see Table A.5).

**Figure 6:**
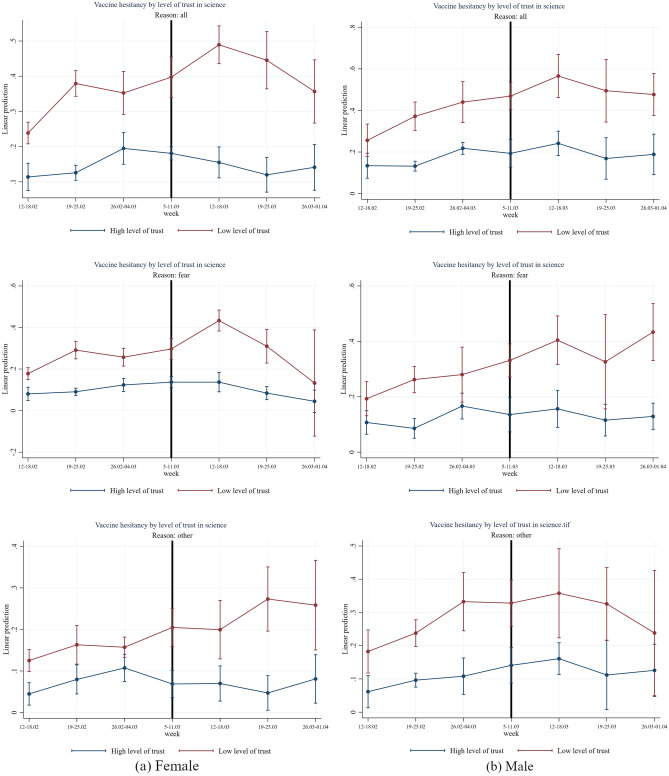
Effect of AstraZeneca ban by level of trust in science and gender. *Note*: The figure shows estimation coefficients for the impact of the week dummies before and after the introduction of the AstraZeneca ban for weeks y = − 4 until y =  + 2 based on an event study regression according to Eq. (1), separately for male and female. The vertical line indicates the week prior to the week of the AstraZeneca ban. The upper graphs show estimation coefficients for all reasons for vaccine hesitancy, the middle graphs show estimation coefficients for fear as main reason for vaccine hesitancy, and the graphs at the bottom, show regression coefficients for other reasons (mainly conspiracy reasons) for vaccine hesitancy. The red line shows the results for the group with a low level of trust in science (having a score within the first or second country-specific terciles on a 1–10 Likert scale) and the blue line shows the results for the group with high level of trust (having a score within the third country-specific tercile on a 1–10 Likert scale). The vertical bars show the upper and lower bounds of the 95 percent confidence intervals of the estimation coefficients.

**Figure 7:**
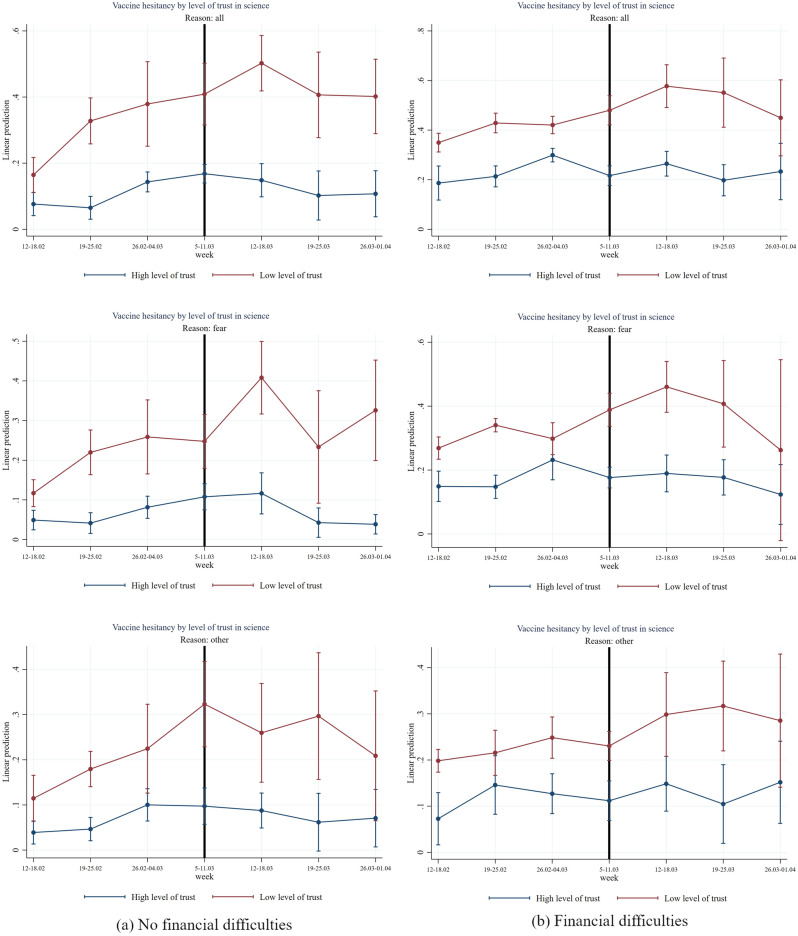
Effect of AstraZeneca ban by level of trust in science and of financial difficulties. *Note*: The figure shows estimation coefficients for the impact of the week dummies before and after the introduction of the AstraZeneca ban for weeks y = − 4 until y =  + 2 based on an event study regression according to Eq. (1), separately by level of financial difficulties (See section “Methods”). The vertical line indicates the week prior to the week of the AstraZeneca ban. The upper graphs show estimation coefficients for all reasons for vaccine hesitancy, the middle graphs show estimation coefficients for fear as main reason for vaccine hesitancy, and the graphs at the bottom, show regression coefficients for other reasons (mainly conspiracy reasons) for vaccine hesitancy. The red line shows the results for the group with a low level of trust in science (having a score within the first or second country-specific terciles on a 1–10 Likert scale) and the blue line shows the results for the group with high level of trust (having a score within the third country-specific tercile on a 1–10 Likert scale). The vertical bars show the upper and lower bounds of the 95 percent confidence intervals of the estimation coefficients.

**Figure 8:**
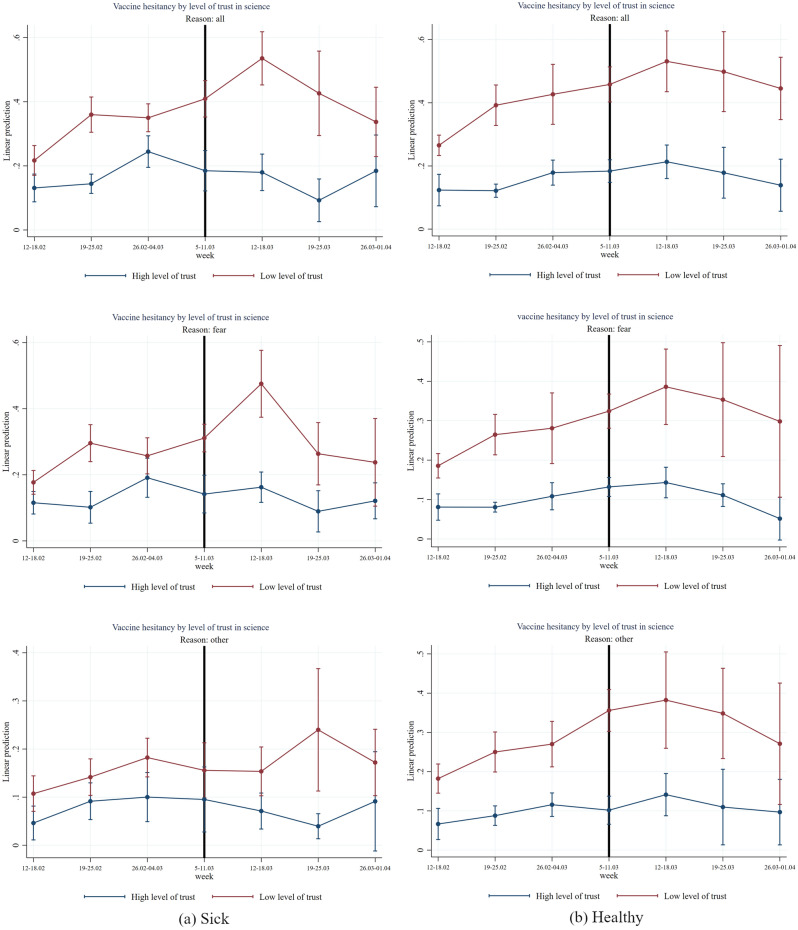
Effect of AstraZeneca ban by level of trust in science and of health status. *Note*: The figure shows estimation coefficients for the impact of the week dummies before and after the introduction of the AstraZeneca ban for weeks y = − 4 until y =  + 2 based on an event study regression according to Eq. (1) (see section “Methods”), separately by health status. The vertical line indicates the week prior to the week of the AstraZeneca ban. The upper graphs show estimation coefficients for all reasons for vaccine hesitancy, the middle graphs show estimation coefficients for fear as main reason for vaccine hesitancy, and the graphs at the bottom, show regression coefficients for other reasons (mainly conspiracy reasons) for vaccine hesitancy. The red line shows the results for the group with a low level of trust in science (having a score within the first or second country-specific terciles on a 1–10 Likert scale) and the blue line shows the results for the group with high level of trust (having a score within the third country-specific tercile on a 1–10 Likert scale). The vertical bars show the upper and lower bounds of the 95 percent confidence intervals of the estimation coefficients.

**Figure 9:**
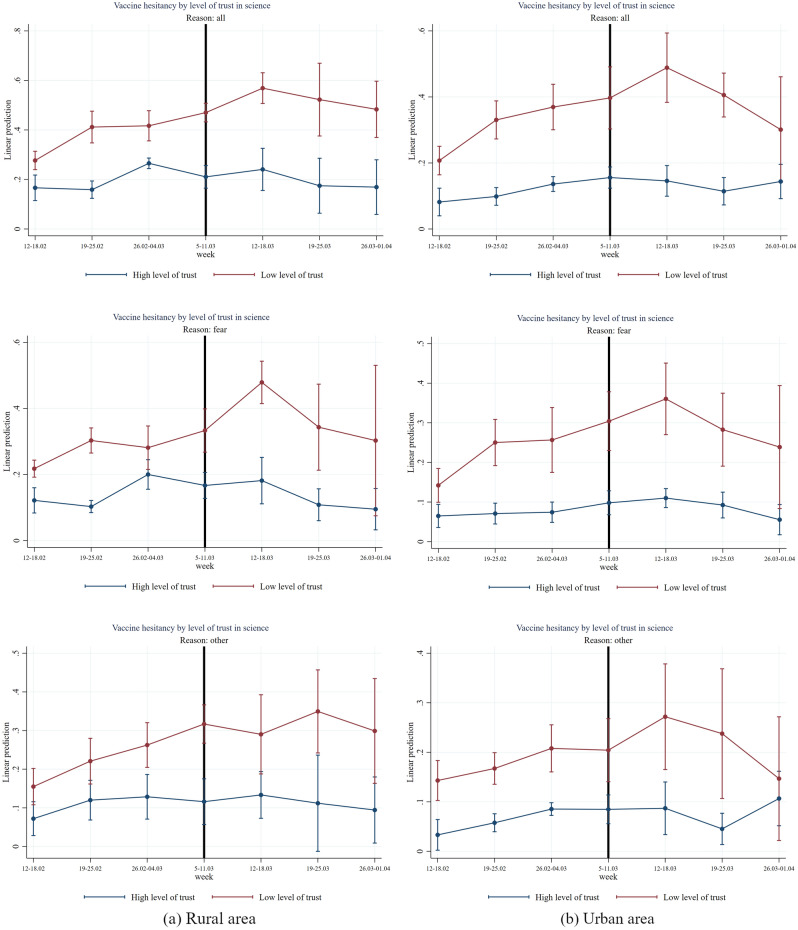
Effect of AstraZeneca ban by level of trust in science and rural/urban areas. *Note*: The figure shows estimation coefficients for the impact of the week dummies before and after the introduction of the AstraZeneca ban for weeks y = − 4 until y =  + 2 based on an event study regression according to Eq. (1) (see section “Methods”), separately by urban/rural areas. The vertical line indicates the week prior to the week of the AstraZeneca ban. The upper graphs show estimation coefficients for all reasons for vaccine hesitancy, the middle graphs show estimation coefficients for fear as main reason for vaccine hesitancy, and the graphs at the bottom, show regression coefficients for other reasons (mainly conspiracy reasons) for vaccine hesitancy. The red line shows the results for the group with a low level of trust in science (having a score within the first or second country-specific terciles on a 1–10 Likert scale) and the blue line shows the results for the group with high level of trust (having a score within the third country-specific tercile on a 1–10 Likert scale). The vertical bars show the upper and lower bounds of the 95 percent confidence intervals of the estimation coefficients.

**Figure 10:**
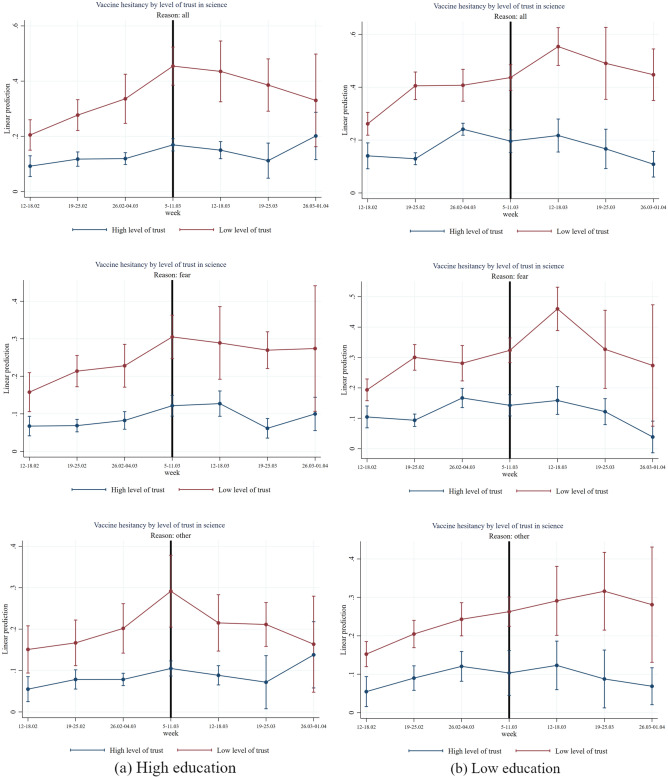
Effect of AstraZeneca ban by level of trust in science and high/low education. *Note*: The figure shows estimation coefficients for the impact of the week dummies before and after the introduction of the AstraZeneca ban for weeks y = − 4 until y =  + 2 based on an event study regression according to Eq. (1) (see section “Methods”), separately by education. The vertical line indicates the week prior to the week of the AstraZeneca ban. The upper graphs show estimation coefficients for all reasons for vaccine hesitancy, the middle graphs show estimation coefficients for fear as main reason for vaccine hesitancy, and the graphs at the bottom, show regression coefficients for other reasons (mainly conspiracy reasons) for vaccine hesitancy. The red line shows the results for the group with a low level of trust in science (having a score within the first or second country-specific terciles on a 1–10 Likert scale) and the blue line shows the results for the group with high level of trust (having a score within the third country-specific tercile on a 1–10 Likert scale). The vertical bars show the upper and lower bounds of the 95 percent confidence intervals of the estimation coefficients.

In Figs. 6, 7, 8, 9 and 10 we report the results of the heterogeneous effect of AstraZeneca suspensions among categories found to be generally more hesitant to vaccinate. In Fig. 6, we show that the suspension caused a jump in hesitancy especially for both women and men with low trust in science. Interestingly—and in line with findings from the European Medical Agency that women were more affected by AstraZeneca complications than men –, fear of vaccine seems to be the most relevant reason for this jump in hesitancy after the suspension for women (two-sided *p*-value = 0.036, see Table A.6). For men in the low trust group, other reasons, such as conspiracy reasons seem quantitatively even more important. (not statistically significant at conventional levels (see Table A.6)).

In Fig. 7, we show that people with low trust in science and financial difficulties have significantly much higher vaccine hesitancy rates compared to people with high trust in science who also suffer from financial difficulties and for “fear” reasons (two-sided *p*-value 0.059, see Table A.6). The significant difference between low trust and high trust people with financial difficulties amounts to about 25 percentage points before the suspension of AstraZeneca (two-sided *p*-value = 0.000) and increases further to roughly 30 percentage points in the week after the AstraZeneca suspension (two-sided *p*-value = 0.000).

Figure 8 shows results for people with low trust in science compared to high trust in science differentiated by their health status. Particularly, for people with poor health and low trust in science the AstraZeneca suspension significantly increases the vaccine hesitancy in the week after the suspension (two-sided *p*-value = 0.048, see Table A.6). Interestingly, fear of vaccine is particularly important for the jump in vaccine hesitancy for the low trust group of sick people in the week after suspension (two-sided *p*-value = 0.062, see Table A.6) whereas other reasons for vaccine hesitancy like conspiracy beliefs are less relevant for hesitancy after the week after suspension of AstraZeneca and not statistically significant at conventional levels (see Table A.6).

Figure 9 displays the results for people with low trust in science compared to high trust in science differentiated by rural vs. urban regions. Results for both regions show a similar pattern than before: (i) substantial differences between low trust and high trust groups (two-sided *p*-values < 0.000), (ii) a significant and substantial impact of the AstraZeneca suspension in the week after the suspension in the group of people with low trust in science (two-sided *p*-value = 0.000) and (iii) a significant jump in the week after the AstraZeneca suspension that mostly stems from the group of people with low trust that state fear of vaccine as main reason for hesitancy (two-sided *p*-value = 0.044, see Table A.6). An exemption regarding the latter results are urban regions where we also see a jump in the conspiracy beliefs as reason for vaccine hesitancy in the low trust group in the week after AstraZeneca suspension but not statistically significant at conventional levels (see Table A.6).

Finally, Fig. 10 shows that education seems to have a protective role for vaccine hesitancy. Indeed, we found that even people with low trust in science do not display an increased vaccine hesitancy after the suspension of AstraZeneca, if highly educated. On the contrary, for low educated, the effect of low trust is higher and determines a significant increase in vaccine hesitancy after the suspension (two-sided *p*-value = 0.033, see Table A.6) and especially for “fear” reasons (two-sided *p*-value = 0.031, see Table A.6). Table A.5 and Table A.6 in the Appendix present tabular representations of coefficients standard errors and *p*-values for Figs. 5, 6, 7, 8, 9 and 10.

## Discussion

COVID-19 vaccine hesitancy is substantial around the world and has increased over time^27,28^. While there is a broad literature that analyzes the reasons for vaccine hesitancy, the existing evidence on the role of trust for vaccine hesitancy is limited even though many studies point to the importance of trust in this decision. These studies also acknowledge the need for future research, explicitly considering the role of trust in vaccine hesitancy^17,29^.

In this paper, we fill this gap by analyzing the role of trust in various dimensions and COVID-19 vaccine hesitancy. We found that higher trust in science is negatively correlated with COVID-19 vaccine hesitancy. This result is consistent with recent findings from Travis et al. who found for the US that trust in science was one of the strongest predictors of vaccine intention^30^ and are in line with results from Tan et al. who found for Singapore that trust in formal sources of information (e.g. from government sources) reduces vaccine hesitancy^17^. Higher trust in social media is also positively correlated with vaccine hesitancy in EU countries. Further research is necessary to understand the causal mechanism behind these correlations. However, results are in line with findings from studies showing that the belief in conspiracy theories and misinformation about the vaccine—which are both largely diffused through social media—are significant predictors of vaccine hesitancy^17,31,32^.

Moreover, we found that people using social media as the main source of information are much more vaccine hesitant compared to people who use classical media sources (TV, Press and Radio) especially because of conspiracy beliefs (COVID risk is exaggerated and/or COVID does not exist). This finding is important and might be explained by two symmetric reasons. On one hand, traditional media sources are more often run by public institutions than social media, and therefore the information published through classical media sources might be more likely to be associated with official opinions that are less critical about vaccination. On the other hand, the diffusion of vaccine-related misinformation on social media might exacerbate the levels of vaccine hesitancy and hamper progress toward vaccine-induced herd immunity as argued by Muric et al. based on a description and assessment of data posts on Twitter^33^. Thus, further research is necessary to understand why media sources are so relevant for vaccine hesitancy and public policy should address the media-specific causes and mechanisms when allocating pro-vaccine campaigns.

Relatedly, and relevant for targeted policy interventions that aim to increase vaccine take-up, we also found that being younger, being better educated, having no financial difficulties and having had experience with COVID-19 directly or among close persons are all associated with higher trust in science compared to their counterparts, whereas unemployment status is correlated with high trust in social media.

Finally, we found that the AstraZeneca temporary suspension was associated with an increase in hesitancy by more than 4 percentage points in the week just after the suspension, especially among those who are worried that the vaccine damages their health. Among people with low trust in science, the suspension caused an increase of approximately 10 percentage points in vaccine hesitancy. The effect of the ban on vaccine hesitancy is found to be larger for people with low trust in science and living in rural areas, women, people with financial difficulties, and reporting bad health. Moreover, we found that education played a protective role among people with low trust in science. All these findings suggest that trust is a key determinant of vaccine hesitancy that generates both a direct effect and an interaction effect along with individual-level factors traditionally associated with a higher risk of vaccine hesitancy. In terms of policy implications, this suggests that pro-vaccine campaigns aiming to increase the trust in public and health authorities could be successfully targeted toward these groups with a high baseline risk of hesitancy. Interventions might also address the physicians as key communicator within the health system. E.g. Maurer (2009) and Schmitz and Wübker (2011), highlight the role of physician agency and quality in the context of influenza vaccination and find evidence that physicians might play a major role in the vaccination decision of many people^34,35^. The role of primary care physicians in the COVID-19 vaccination decision in the context and interplay of the various dimensions of trust could be a useful area of future research.

The study faces some limitations. First, the analytical methods to assess the determinants of vaccine hesitancy and the effect of trust were cross-sectional in nature—with the notable exception of the event-study analysis of the AstraZeneca ban—and do not allow a causal interpretation of the main findings. However, the available data set is very rich which allows us to control for a number of individual-level variables potentially associated with both vaccine hesitancy and trust. This allays endogeneity concerns in a substantial way. Moreover, our results are in line with other studies that analyzed COVID-19 vaccine hesitancy that used different methods and additionally provide some interesting insight regarding heterogeneous effects. Second, the interpretation of the different reasons (“fear” and “others—mainly conspiracy beliefs”) and the determinants of the different reasons for refusing the vaccine should be done cautiously and can only be speculative, as we cannot perfectly distinguish between the reasons for not vaccinating. For instance, according to Douglas et al. (2017) a conspiracy belief is “an attempt to explain the ultimate cause of an event or situation as a secret, often sinister, plot by a covert alliance”^36^. While conspiracy beliefs, as defined above are a likely reason for not vaccinating among people answering that “COVID-19 risk does not exist” or “COVID-19 risk is exaggerated”, “conspiracy” is not explicitly mentioned in these statements. Moreover, as fear of vaccination might be a result also of a conspiracy belief (e.g. people think that governments want to track people) these concepts might partially intersect each other. Therefore, the constructs “fear reasons” and “conspiracy reasons” might be not entirely separable in our data. Third, the sampling design via the snowball system produces a sample that might not be entirely representative of the European population. However, the sample was weighted based on gender, age, education and urbanization levels which allows us to draw credible inference on the relationship between trust and vaccine hesitancy that can be easily generalized to the European population. Finally, our results represent the pandemic situation in spring 2021 and the assessment of the AstraZeneca ban is a special case of a health system-level disorder. The pandemic situation regarding COVID-19 changed over time; evidence on the effectiveness and side effects of vaccines are constantly evolving. This limitation in the external validity should be taken into account when interpreting the results.

Having these limitations in mind, the results of the study provide valuable implications for our understating of the role of trust in immunization choices, and for the design of global health policies aimed to fight vaccine hesitancy and obtain long-term herd immunity of the population.

## Methods

### Data description

We use the Eurofound’s Living, Working and Covid-19 online survey for the round 3 collected between the 12 February to 1 April 2021. It is a repeated cross-section survey collected on a weekly basis and involving around 35,000 individuals living in the European Union. The survey was allocated via snowball sampling method reaching out to stakeholders in the member states and social media advertisements^37^. The sample is weighted based on age, gender, education, and urbanization levels, to obtain representative data of the demographic profile of the European Union as a whole and of the member states. The survey collects information on five topics: (a) trust scores on 10 point Likert scale from 1 “Do not trust at all” to 10 “Trust completely” for the following dimensions: social media, government, science, social media, police, EU, health care system, pharmaceutical firms, people; (b) COVID-19 immunization intentions and take-up along with reasons for hesitancy (concerns about safety, COVID-19 risk is exaggerated, COVID-19 does not exist, etc.); (c) media use (TV, radio, social media, etc.); (d) socio-economic and demographic information such as age, financial difficulties, education, living area (rural, urban, etc.), employment status; (e) health and COVID-19 information such as whether the respondent tested positive for COVID-19 , whether she had someone close to her tested positive or died from COVID-19 or another causes along with self-reported health status on a 5 point ordinal scale from “very good” to “very bad”. Self-assessed health (SAH) is a widely used tool to estimate population health and it has been found to be highly correlated with objective health measures such as the use of physician services and, more importantly, mortality outcomes^38,39^. Table A.7 in the Appendix reports descriptive statistics of the variables used in this paper.

In the second week of March 2021, a number of European countries officially announced that they were temporarily suspending the use of the AstraZeneca vaccine against COVID-19 after it had been associated to very rare cases of thrombosis and pulmonary embolism (compare for more details on AstraZeneca suspension ECDC)^24,26^. Austria announced on March 7 that it would suspend a single batch of the vaccine. Denmark, Norway, and Iceland made a similar decision on March 11, and by March 16, 2021, 17 EU countries had suspended use of the vaccine. Because of reports of adverse reactions to the vaccine, the EMA launched a formal investigation into known cases of thrombosis and embolism associated with the vaccine on March 11, 2021. These investigations and the suspension of the vaccine made headlines around the world^25^. However, on March 18, 2021, the EMA Safety Committee concluded that the benefits still outweighed the risks despite a possible association with rare blood clots with low blood platelets^40^.

In this study we compare intentions to vaccinate on a weekly basis from February to April 2021 before and after the week in which suspensions were concentrated in Europe (12–18 March 2021) using an event-study approach—explained below –, as concerns about side effects might be significantly associated with vaccine hesitancy in the context of COVID-19 vaccines.

### Empirical model

The analysis on determinants of COVID-19 vaccine hesitancy (Table A.1) is based on a multivariate linear probability model estimated with ordinary least squares method. The estimations are weighted based on age, gender, education, and urbanization levels, to obtain representative data of the demographic profile of the European Union as a whole and of the member states. The model includes country and week fixed effects to consider time-invariant unobservable characteristics varying at country level, and time trends in immunization choices, respectively. Our outcome variable is the intention to vaccinate based on the question “How likely or unlikely is it that you will take the COVID-19 vaccine when it becomes available to you?”. Following other studies on COVID-19 vaccine hesitancy, i.e. (Lazarus et al., 2021)^2^, we coded as vaccine hesitant individuals who replied “Rather unlikely or very unlikely”. We prefer to measure the intentions to vaccinate rather than the actual take-up because it is more adherent with the classical definition of vaccine hesitancy and because the latter might be influenced by the supply of vaccines in the area. Formally, we estimate:

1 $$VH_{i} = \alpha_{0} + \alpha_{1} Trust_{i} + \alpha_{2} Infsource_{i} + \alpha_{3} SESD_{i} + \alpha_{4} Health_{i} + \gamma_{w} + \delta_{c} + \varepsilon_{i}$$

where the dependent variable vaccine hesitancy $VH_{i}$ is a dummy variable that indicates whether a person i does not intent to vaccinate against Covid-19. $Trust_{i}$ is a vector of the trust scores (from 0 “no trust at all” to 10 “trust completely”) in the following dimensions: social media, government, science, social media, police, EU, health care system, pharmaceutical firms, people. $Infsource_{i}$ is a vector of dummy variables indicating the main source of media use (e.g. press, TV, radio, social media). $SESD_{i}$ includes various socioeconomic (e.g. education, employment status) and sociodemographic (e.g. age, gender) variables. $Health_{i}$ includes self-assessed health but also experience with COVID-19 (own infection, infection of a relative, death of a relative due to COVID-19 but also due to other reasons). $\gamma_{w}$ and $\delta_{c}$ are respectively week and country fixed effects and control for differences in vaccine hesitancy levels across weeks between March and April 2021 and countries. The $\alpha$ s are parameters and $\varepsilon_{i}$ is the normal error term. Standard errors are clustered at the country level. Estimates of Eq. (1) are displayed in Figs. 1, 2, and Table A.1. Additional results reported in Table A.2 are based on Eq. (1) in which the trust variables are replaced by the following trust variable with four categories: high trust in science only, high trust in social media only, low trust in both, high trust in both. An individual i is categorized as having high trust when her score is within the third country-specific tercile, and as having low trust when her score is within the first and second country-specific terciles.

The analysis of trust formation is performed based on an OLS model, as follows:

2 $$Trust_{i} = \beta_{0} + \beta_{1} Infsource_{i} + \beta_{2} SESD_{i} + \beta_{3} Health_{i} + \gamma_{w} + \delta_{c} + \varepsilon_{i}$$

where Eq. (2) is estimated for each trust variable (news, science, social media, police, government, EU, health system, pharmaceutical firms, people). Similarly to Eq. (1), the estimations of Eq. (2) are weighted to obtain representative data of the demographic profile of the European Union as a whole and of the member states. Standard errors are clustered at the country level. Estimates are reported in Fig. 3 and Table A.3.

The association between the AstraZeneca ban and vaccine hesitancy is measured based on Eq. (1) in which we look specifically at the variable week, $\gamma_{w}$ in an event study design^41,42^. In this way, we compare immunization intentions in the weeks before and after the week of suspension (12–18 March 2021). An event study design allows transparently assessing pre-existing trends and post-suspension dynamics in vaccine hesitancy.

Eventually, to estimate the heterogeneous response to the ban, we first measure the association between the ban of AstraZeneca, the levels of trust in science, and vaccine hesitancy. We modify Eq. (1) by adding an interaction term between the week and the level of trust in science. High (low) trust in science in week w is defined as having a score within the third (first and second) country-specific tercile(s) in week w. Second, we estimate these associations separately for different characteristics: gender, financial difficulties, health status, urban/rural, and educational attainment.

Results of the association between the AstraZeneca ban with vaccine hesitancy are reported in Table A.4 to Table A.6 and graphically displayed in Figs. 4, 5, 6, 7, 8, 9 and 10.

## Supplementary Information

Supplementary Information.

## Data Availability

In this paper, we use survey data compiled by Eurofound. The data sample used cannot be published on the website as these are partly proprietary data. However, researchers can apply at Eurofound to obtain access to the data. It is expected that reasonable applications including projects that aim to replicate this paper will be accepted by Eurofound. The data application process includes a short informal request for data use at Eurofound (contact: information@eurofound.europa.eu). Further information regarding the services provided by Eurofound can be found here: https://www.eurofound.europa.eu/surveys/living-working-and-covid-19-e-survey and regarding general data availability of their survey data here: https://www.eurofound.europa.eu/surveys/about-eurofound-surveys/data-availability

## References

[1] EMA. *Safety of COVID-19 vaccines*, https://www.ema.europa.eu/en/human-regulatory/overview/public-health-threats/coronavirus-disease-covid-19/treatments-vaccines/vaccines-covid-19/safety-covid-19-vaccines (2022).

[2] Lazarus JV (2021). A global survey of potential acceptance of a COVID-19 vaccine. Nat. Med..

[3] ECDC. *COVID - 19 Country overviews - Country overview report*, https://covid19-country-overviews.ecdc.europa.eu/ (2022).

[4] WHO. *Ten threats to global health in 2019*, https://www.who.int/news-room/spotlight/ten-threats-to-global-health-in-2019 (2019).

[5] Horne Z, Powell D, Hummel JE, Holyoak KJ (2015). Countering antivaccination attitudes. Proc. Natl. Acad. Sci..

[6] ECDC. *Monthly measles and rubella monitoring*, https://www.ecdc.europa.eu/en/rubella/surveillance-and-disease-data/monthly-measles-rubella-monitoring-reports (2022).

[7] Dincer O, Gillanders R (2021). Shelter in place? Depends on the place: Corruption and social distancing in American states. Soc. Sci. Med..

[8] Hornsey MJ, Finlayson M, Chatwood G, Begeny CT (2020). Donald Trump and vaccination: The effect of political identity, conspiracist ideation and presidential tweets on vaccine hesitancy. J. Exp. Soc. Psychol..

[9] Ward JK (2020). The French public's attitudes to a future COVID-19 vaccine: The politicization of a public health issue. Soc. Sci. Med..

[10] Chang LV (2018). Information, education, and health behaviors: Evidence from the MMR vaccine autism controversy. Health Econ..

[11] Yaqub O, Castle-Clarke S, Sevdalis N, Chataway J (2014). Attitudes to vaccination: A critical review. Soc. Sci. Med..

[12] Machingaidze S, Wiysonge CS (2021). Understanding COVID-19 vaccine hesitancy. Nat. Med..

[13] Gerretsen P (2021). Individual determinants of COVID-19 vaccine hesitancy. PLoS ONE.

[14] Wang Y, Liu Y (2022). Multilevel determinants of COVID-19 vaccination hesitancy in the United States: A rapid systematic review. Prevent. Med. Rep..

[15] Arrow K (1972). Gifts and exchanges. Philos. Public Aff..

[16] Putnam RD (1993). Making Democracy Work.

[17] Tan M, Straughan PT, Cheong G (2022). Information trust and COVID-19 vaccine hesitancy amongst middle-aged and older adults in Singapore: A latent class analysis Approach. Soc. Sci. Med..

[18] Sturgis P, Brunton-Smith I, Jackson J (2021). Trust in science, social consensus and vaccine confidence. Nat. Hum. Behav..

[19] Betsch C (2018). Beyond confidence: Development of a measure assessing the 5C psychological antecedents of vaccination. PLoS ONE.

[20] Roozenbeek J (2020). Susceptibility to misinformation about COVID-19 around the world. R. Soc. Open Sci..

[21] Freeman D (2022). Coronavirus conspiracy beliefs, mistrust, and compliance with government guidelines in England. Psychol Med.

[22] Greinacher A (2021). Thrombotic Thrombocytopenia after ChAdOx1 nCov-19 Vaccination. N. Engl. J. Med..

[23] Scully M (2021). Pathologic Antibodies to Platelet Factor 4 after ChAdOx1 nCoV-19 Vaccination. N. Engl. J. Med..

[24] Deiana C, Geraci A, Mazzarella G, Sabatini F (2022). Perceived risk and vaccine hesitancy: Quasi-experimental evidence from Italy. Health Econ..

[25] Petersen MB, Jørgensen F, Lindholt MF (2022). Did the European suspension of the AstraZeneca vaccine decrease vaccine acceptance during the COVID-19 pandemic?. Vaccine.

[26] Agosti F (2022). Information and vaccine hesitancy: Evidence from the early stage of the vaccine roll-out in 28 European countries. PLoS ONE.

[27] Lin, C., Tu, P. & Beitsch, L. M. Confidence and Receptivity for COVID-19 Vaccines: A Rapid Systematic Review. *Vaccines* **9** (2021). https://mdpi-res.com/d_attachment/vaccines/vaccines-09-00016/article_deploy/vaccines-09-00016-v2.pdf?version=1609734577 10.3390/vaccines9010016 PMC7823859 33396832

[28] Aw, J., Seng, J. J., Seah, S. S. & Low, L. L. COVID-19 Vaccine Hesitancy—A Scoping Review of Literature in High-Income Countries. *Vaccines* **9** (2021). https://mdpi-res.com/d_attachment/vaccines/vaccines-09-00900/article_deploy/vaccines-09-00900-v2.pdf?version=1629357218 10.3390/vaccines9080900 PMC8402587 34452026

[29] Schwarzinger M, Watson V, Arwidson P, Alla F, Luchini S (2021). COVID-19 vaccine hesitancy in a representative working-age population in France: A survey experiment based on vaccine characteristics. Lancet Public Health.

[30] Travis J, Harris S, Fadel T, Webb G (2021). Identifying the determinants of COVID-19 preventative behaviors and vaccine intentions among South Carolina residents. PLoS ONE.

[31] Carrieri V, Madio L, Principe F (2019). Vaccine hesitancy and (fake) news: Quasi-experimental evidence from Italy. Health Econ..

[32] Loomba S, de Figueiredo A, Piatek SJ, de Graaf K, Larson HJ (2021). Measuring the impact of COVID-19 vaccine misinformation on vaccination intent in the UK and USA. Nat. Hum. Behav..

[33] Muric G, Wu Y, Ferrara E (2021). COVID-19 vaccine hesitancy on social media: Building a public twitter data set of antivaccine content, vaccine misinformation, and conspiracies. JMIR Public Health Surveill..

[34] Maurer J (2009). Who has a clue to preventing the flu? Unravelling supply and demand effects on the take-up of influenza vaccinations. J. Health Econ..

[35] Schmitz H, Wübker A (2011). What determines influenza vaccination take-up of elderly Europeans?. Health Econ..

[36] Douglas KM, Sutton RM, Cichocka A (2017). The psychology of conspiracy theories. Curr. Dir. Psychol. Sci..

[37] Eurofound. Living, working and COVID-19: Mental health and trust decline across EU as pandemic enters another year. (2021).

[38] Idler EL, Benyamini Y (1997). Self-rated health and mortality: A review of twenty-seven community studies. J. Health Soc. Behav..

[39] Miilunpalo S, Vuori I, Oja P, Pasanen M, Urponen H (1997). Self-rated health status as a health measure: The predictive value of self-reported health status on the use of physician services and on mortality in the working-age population. J. Clin. Epidemiol..

[40] ECDC. Overview of the implementation of COVID-19 vaccination strategies and vaccine deployment plans in the EU/EEA. (European Centre for Disease Prevention and Control, https://www.ecdc.europa.eu/sites/default/files/documents/Overview-implementation-COVID-19-vaccination-strategies-vaccine-deployment-plans.pdf, 2022).

[41] MacKinlay AC (1997). Event studies in economics and finance. J. Econ. Lit..

[42] Angrist JD, Pischke J (2008). Mostly harmless econometrics: An empiricist's companion.

