# Supplementary material for: Trust and COVID-19 vaccine hesitancy
Source: Sci Rep. 2023 Jun 7;13:9245. doi: 10.1038/s41598-023-35974-z (PMC10245358; doi:10.1038/s41598-023-35974-z)
Supplement: Supplementary file 1 — Supplementary Information. [file 41598_2023_35974_MOESM1_ESM.docx]

Trust and COVID-19 vaccine hesitancy

| **Vincenzo Carrieri** | **Sophie Guthmuller*** | **Ansgar Wübker** |
| --- | --- | --- |
| Department of Political and Social Sciences, University of Calabria; RWI-Leibniz Institute for Economic Research; IZA | Vienna University of Economics and Business; RWI-Leibniz Institute for Economic Research | Hochschule Harz; RWI-Leibniz Institute for Economic Research |

* Corresponding author: sophie.guthmuller@wu.ac.at

## Appendix

**Table A.1: Determinants of COVID-19 vaccine hesitancy**

|  |  | (1) | | (2)  Vaccine hesitancy:  fear | | (3)  Vaccine hesitancy: other reasons | |
| --- | --- | --- | --- | --- | --- | --- | --- |
| Variables | | Vaccine hesitancy:  all reasons | |  |  |  |  |
| Trust | … in news media | -0.023^***^ | (0.003) | -0.016^***^ | (0.003) | -0.016^***^ | (0.002) |
|  | … in police | -0.003 | (0.002) | -0.002 | (0.002) | -0.006^*^ | (0.003) |
|  | … in government | -0.007^**^ | (0.003) | -0.007^***^ | (0.001) | -0.000 | (0.003) |
|  | … in EU | -0.010^***^ | (0.003) | -0.005^**^ | (0.002) | -0.010^***^ | (0.002) |
|  | … in healthcare system | -0.008^***^ | (0.002) | -0.004^**^ | (0.001) | -0.011^***^ | (0.004) |
|  | … in social media | 0.020^***^ | (0.003) | 0.016^***^ | (0.003) | 0.012^***^ | (0.004) |
|  | … in science | -0.031^***^ | (0.005) | -0.025^***^ | (0.007) | -0.016^***^ | (0.005) |
|  | … in pharmaceutical firms | -0.029^***^ | (0.003) | -0.022^***^ | (0.003) | -0.016^***^ | (0.003) |
|  | … in people | 0.008^***^ | (0.001) | 0.005^**^ | (0.002) | 0.008^***^ | (0.002) |
| Main source of information | TV | ref |  | ref |  | ref |  |
|  | Press | -0.007 | (0.018) | 0.001 | (0.012) | -0.011 | (0.015) |
|  | Radio | 0.008 | (0.024) | 0.010 | (0.013) | 0.002 | (0.018) |
|  | Social media | 0.058^**^ | (0.028) | 0.011 | (0.032) | 0.057^***^ | (0.020) |
|  | Other media | 0.112^***^ | (0.029) | 0.074^***^ | (0.026) | 0.101^***^ | (0.027) |
|  | Female | -0.015^*^ | (0.007) | -0.008 | (0.008) | -0.041^***^ | (0.007) |
| Age | 18 - 29 | 0.047^*^ | (0.027) | 0.046 | (0.035) | 0.082^***^ | (0.025) |
|  | 30 - 49 | 0.060^**^ | (0.023) | 0.041^*^ | (0.022) | 0.068^***^ | (0.022) |
|  | 50 – 64 | 0.027 | (0.016) | 0.016 | (0.018) | 0.012 | (0.017) |
|  | 65+ | ref |  | ref |  | ref |  |
| Region | The open countryside | 0.105^***^ | (0.022) | 0.080^***^ | (0.025) | 0.044^**^ | (0.016) |
|  | A village/small town | 0.046^***^ | (0.010) | 0.037^***^ | (0.005) | 0.031^**^ | (0.014) |
|  | A medium to large town | 0.027 | (0.017) | 0.019 | (0.020) | 0.007 | (0.006) |
|  | A city or city suburb | ref |  | ref |  | ref |  |
| Household's ability to make ends meet | With great difficulty | ref |  | ref |  | ref |  |
|  | With difficulty | -0.044^**^ | (0.018) | -0.046^**^ | (0.022) | -0.018 | (0.016) |
|  | With some difficulty | -0.041^*^ | (0.024) | -0.056^**^ | (0.027) | 0.016 | (0.019) |
|  | Fairly easily | -0.036^*^ | (0.021) | -0.064^***^ | (0.021) | 0.022 | (0.015) |
|  | Easily | -0.068^***^ | (0.024) | -0.088^***^ | (0.027) | 0.010 | (0.014) |
|  | Very easily | -0.050^**^ | (0.021) | -0.089^***^ | (0.028) | 0.015 | (0.015) |
| Employment status | Employee | ref |  | ref |  | ref |  |
|  | Self-employed | 0.018 | (0.014) | -0.028^**^ | (0.013) | 0.035^***^ | (0.012) |
|  | Unemployed | 0.025 | (0.022) | 0.017 | (0.018) | 0.024 | (0.018) |
|  | Retired | -0.014 | (0.030) | -0.007 | (0.026) | -0.018 | (0.025) |
|  | Other | 0.005 | (0.028) | -0.008 | (0.020) | -0.011 | (0.020) |
| Edu-cation | Primary | ref |  | ref |  | ref |  |
|  | Secondary | 0.021 | (0.032) | 0.042^**^ | (0.018) | 0.009 | (0.018) |
|  | Tertiary | -0.002 | (0.029) | 0.030^*^ | (0.016) | -0.005 | (0.016) |
| COVID-19 | Tested positive | 0.025^***^ | (0.006) | 0.029 | (0.018) | 0.009 | (0.019) |
|  | Someone close to you tested positive | -0.013 | (0.011) | 0.001 | (0.009) | -0.017^**^ | (0.007) |
|  | Someone close to you died | -0.019 | (0.016) | -0.032^**^ | (0.013) | -0.004 | (0.014) |
|  | Someone close to you died from another cause | 0.052^***^ | (0.011) | 0.036^**^ | (0.014) | 0.047^***^ | (0.008) |
| Self-perceived health | Very good | ref |  | ref |  | ref |  |
|  | Good | -0.081^***^ | (0.006) | -0.041^***^ | (0.006) | -0.065^***^ | (0.009) |
|  | Fair | -0.119^***^ | (0.012) | -0.056^***^ | (0.013) | -0.117^***^ | (0.017) |
|  | Bad | -0.093^***^ | (0.029) | -0.001 | (0.038) | -0.136^***^ | (0.029) |
|  | Very bad | -0.076 | (0.047) | -0.012 | (0.076) | -0.132^***^ | (0.042) |
| N |  | 35,757 |  | 35,757 |  | 35,757 |  |
| R² |  | 0.3752 |  | 0.2557 |  | 0.2775 |  |

Note: The table reports estimates of eq.1 for the displayed variables (see section Methods). Weekly and country fixed effects are included. Clustered standard errors at the country level in are parentheses, * p < 0.1, ** p < 0.05, *** p < 0.01

**Table A.2:** **Determinants of COVID-19 vaccine hesitancy: trust categories**

|  |  | (1) | | (2)  Vaccine hesitancy:  fear | | (3)  Vaccine hesitancy: other reasons | |
| --- | --- | --- | --- | --- | --- | --- | --- |
| Variables | | Vaccine hesitancy:  all reasons | |  |  |  |  |
| Trust | High in Science only | -0.015 | (0.020) | -0.022 | (0.015) | 0.001 | (0.021) |
|  | High in Social Media only | 0.098*** | (0.018) | 0.067*** | (0.022) | 0.066*** | (0.012) |
|  | Low in both | 0.163*** | (0.026) | 0.117*** | (0.025) | 0.103*** | (0.017) |
|  | High in both | ref |  | ref |  | ref |  |
| N |  | 35,757 |  | 35,757 |  | 35,757 |  |
| R² |  | 0.2397 |  | 0.1649 |  | 0.1758 |  |

Note: The table reports estimates of eq.1 for the trust categories (see section Methods). Weekly and country fixed effects are included. Clustered standard errors at the country level in are parentheses, * p < 0.1, ** p < 0.05, *** p < 0.01

**Figure A.1: Determinants of trust: Age and COVID-19 related health experiences**

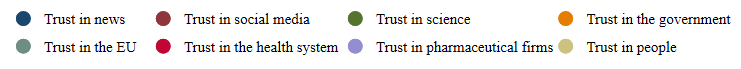


Note: The figure reports the estimates for covid-19 variables and age of eq.2 (see section Methods). The full estimation results are available in Table A.3 in Appendix. * p < 0.1, ** p < 0.05, *** p < 0.01

**Table A.3: Determinants of trust formation**

|  | Trust in … | (1) | (2) | (3) | (4) | (5) | (6) | (7) | (8) |
| --- | --- | --- | --- | --- | --- | --- | --- | --- | --- |
|  |  | News | Science | Government | EU | Health system | Pharma | Social media | People |
| Main source of information.  Ref: TV | Press | -0.120 (0.075) | 0.328^***^ (0.108) | -0.093 (0.164) | 0.410^***^ (0.135) | -0.003 (0.083) | -0.011 (0.084) | -0.236^***^ (0.057) | 0.212^***^ (0.061) |
|  | Radio | -0.176 (0.105) | 0.157 (0.151) | -0.304^**^ (0.127) | 0.185 (0.109) | 0.007 (0.127) | -0.101 (0.175) | -0.321^***^ (0.077) | 0.336^***^ (0.083) |
|  | Social media | -1.348^***^ (0.281) | -0.570^***^ (0.204) | -1.102^***^ (0.341) | -0.748^***^ (0.221) | -0.712^***^ (0.109) | -0.766^***^ (0.195) | 0.538^***^ (0.069) | -0.008 (0.109) |
|  | Other media | -2.321^***^ (0.240) | -0.902^***^ (0.268) | -1.597^***^ (0.357) | -1.372^***^ (0.236) | -1.453^***^ (0.131) | -1.595^***^ (0.190) | -0.844^***^ (0.123) | -0.354^**^ (0.145) |
|  | Female | 0.131^**^ (0.058) | -0.033 (0.062) | 0.143^**^ (0.058) | 0.156 (0.092) | -0.129^**^ (0.065) | 0.072 (0.053) | 0.073^*^ (0.042) | 0.055 (0.076) |
| Age.  Ref: 65+ | 18 - 29 | 0.323^*^ (0.162) | 0.761^**^ (0.277) | -0.213 (0.289) | 0.543^*^ (0.277) | 0.227 (0.154) | 0.439^**^ (0.178) | -0.369^**^ (0.168) | -0.691^***^ (0.135) |
|  | 30 - 49 | -0.085 (0.097) | 0.099 (0.170) | -0.575^**^ (0.211) | -0.214 (0.268) | -0.166 (0.138) | -0.196 (0.160) | -0.555^***^ (0.150) | -0.812^***^ (0.114) |
|  | 50 – 64 | -0.048 (0.097) | -0.114 (0.135) | -0.396^**^ (0.145) | -0.361^*^ (0.188) | -0.158 (0.121) | -0.331^**^ (0.124) | -0.314^**^ (0.117) | -0.389^***^ (0.083) |
| Region.  Ref: A city or city suburb | The open countryside | -0.499^***^ (0.166) | -0.471^***^ (0.088) | -0.484^**^ (0.189) | -0.883^***^ (0.196) | -0.258^**^ (0.126) | -0.262^*^ (0.136) | -0.168 (0.107) | -0.013 (0.184) |
|  | A village/small town | -0.350^***^ (0.086) | -0.341^***^ (0.080) | -0.243^**^ (0.116) | -0.416^***^ (0.091) | -0.160^**^ (0.078) | -0.109 (0.071) | 0.015 (0.063) | -0.093 (0.100) |
|  | A medium to large town | -0.273^***^ (0.065) | -0.208^*^ (0.103) | -0.218^**^ (0.092) | -0.374^***^ (0.086) | -0.076 (0.081) | -0.024 (0.099) | -0.040 (0.073) | -0.038 (0.142) |
| Household's ability to make ends meet.  Ref: With great difficulty | With difficulty | 0.213^**^ (0.103) | 0.265^*^ (0.146) | 0.334^**^ (0.130) | 0.233 (0.140) | 0.313^**^ (0.151) | 0.022 (0.190) | 0.217^***^ (0.060) | 0.258 (0.172) |
|  | With some difficulty | 0.512^***^ (0.137) | 0.358^**^ (0.134) | 0.750^***^ (0.162) | 0.558^**^ (0.242) | 0.495^***^ (0.135) | 0.395^**^ (0.151) | 0.375^***^ (0.114) | 0.554^***^ (0.150) |
|  | Fairly easily | 0.743^***^ (0.121) | 0.811^***^ (0.120) | 1.163^***^ (0.113) | 1.059^***^ (0.173) | 0.787^***^ (0.131) | 0.781^***^ (0.171) | 0.566^***^ (0.070) | 0.647^***^ (0.107) |
|  | Easily | 1.077^***^ (0.211) | 1.094^***^ (0.160) | 1.507^***^ (0.243) | 1.388^***^ (0.305) | 1.140^***^ (0.140) | 1.025^***^ (0.233) | 0.611^***^ (0.129) | 0.951^***^ (0.155) |
|  | Very easily | 1.281^***^ (0.153) | 1.349^***^ (0.217) | 1.813^***^ (0.236) | 1.804^***^ (0.347) | 1.329^***^ (0.155) | 1.434^***^ (0.237) | 0.618^***^ (0.053) | 1.055^***^ (0.108) |

|  | Trust in … | (1) | (2) | (3) | (4) | (5) | (6) | (7) | (8) |
| --- | --- | --- | --- | --- | --- | --- | --- | --- | --- |
|  |  | News | Science | Government | EU | Health system | Pharma | Social media | People |
|  |  |  |  |  |  |  |  |  |  |
| Employment status | Self-employed | -0.376^**^ (0.156) | -0.187 (0.190) | -0.379 (0.262) | -0.160 (0.215) | -0.539^***^ (0.143) | -0.272 (0.172) | -0.190^*^ (0.110) | 0.042 (0.135) |
|  | Unemployed | -0.001 (0.078) | -0.077 (0.119) | 0.031 (0.115) | 0.058 (0.174) | -0.056 (0.124) | 0.182^**^ (0.079) | 0.340^***^ (0.098) | -0.270^***^ (0.074) |
|  | Retired | -0.005 (0.149) | 0.006 (0.154) | 0.062 (0.173) | 0.044 (0.206) | 0.115 (0.124) | 0.091 (0.171) | -0.023 (0.139) | -0.260^**^ (0.115) |
|  | Other | 0.531^***^ (0.114) | 0.256^*^ (0.129) | 0.223 (0.178) | 0.459^***^ (0.112) | 0.347^***^ (0.118) | 0.539^***^ (0.116) | 0.410^***^ (0.121) | 0.035 (0.165) |
| Educa-tion. Ref: Primary | Secondary | 0.052 (0.144) | 0.018 (0.139) | 0.033 (0.180) | 0.234 (0.277) | -0.330^*^ (0.185) | -0.221 (0.192) | 0.017 (0.174) | -0.208^*^ (0.117) |
|  | Tertiary | 0.310^*^ (0.156) | 0.450^**^ (0.209) | 0.359^***^ (0.119) | 0.545^**^ (0.250) | -0.099 (0.181) | 0.068 (0.125) | 0.084 (0.174) | 0.225 (0.132) |
| COVID-19 | Tested positive | -0.034 (0.079) | 0.061 (0.111) | -0.342^**^ (0.123) | -0.402^**^ (0.151) | -0.013 (0.123) | -0.259 (0.207) | 0.019 (0.081) | 0.031 (0.091) |
|  | Someone close to you tested positive | 0.069 (0.063) | 0.167^***^ (0.043) | 0.228^***^ (0.066) | 0.175^***^ (0.042) | 0.191^***^ (0.070) | 0.113^***^ (0.025) | -0.007 (0.057) | 0.173^***^ (0.054) |
|  | Someone close to you died | 0.190 (0.135) | 0.212^*^ (0.116) | 0.104 (0.120) | 0.367^***^ (0.118) | 0.206^*^ (0.113) | 0.367^***^ (0.055) | 0.033 (0.163) | -0.294^**^ (0.141) |
|  | Someone close to you died from another cause | -0.190^***^ (0.048) | -0.091 (0.091) | -0.220^***^ (0.074) | -0.219^**^ (0.082) | -0.265^***^ (0.077) | -0.246^***^ (0.075) | -0.054 (0.048) | -0.034 (0.039) |
| Self-perceived health.  Ref: Very good | Good | 0.209^*^ (0.110) | 0.101 (0.103) | 0.024 (0.127) | 0.043 (0.080) | 0.070 (0.103) | 0.220^*^ (0.116) | 0.040 (0.099) | -0.454^***^ (0.091) |
|  | Fair | 0.123 (0.097) | 0.035 (0.151) | -0.096 (0.106) | -0.030 (0.115) | -0.134 (0.112) | 0.072 (0.130) | 0.025 (0.120) | -0.827^***^ (0.079) |
|  | Bad | -0.014 (0.102) | -0.102 (0.193) | -0.384^**^ (0.154) | -0.044 (0.111) | -0.551^***^ (0.167) | -0.304^***^ (0.077) | -0.087 (0.074) | -1.187^***^ (0.100) |
|  | Very bad | -0.480^*^ (0.257) | -0.929^*^ (0.508) | -0.514^***^ (0.141) | -0.755^***^ (0.161) | -0.847^**^ (0.339) | -0.003 (0.530) | 0.097 (0.319) | -1.977^***^ (0.249) |
| N |  | 35,757 | 35,757 | 35,757 | 35,757 | 35,757 | 35,757 | 35,757 | 35,757 |
| R² |  | .267513 | .2412664 | .2572626 | .2476238 | .2716156 | .2015285 | .0948095 | .1353786 |

Note: The table reports estimates of eq.2 (see section Methods). Country and week fixed effects are included. Clustered standard errors at the country level in are parentheses, * p < 0.1, ** p < 0.05, *** p < 0.01

**Table A.4: The effect of AstraZeneca temporary suspension**

|  | (1)  Vaccine hesitancy: all | | (2)  Vaccine hesitancy:  fear | | (3)  Vaccine hesitancy: other reasons | |
| --- | --- | --- | --- | --- | --- | --- |
| Week: |  |  |  |  |  |  |
| 12-18.02 | -0.026^*^ | (0.013) | -0.014^*^ | (0.008) | -0.015 | (0.021) |
| 19-25.02 | -0.004 | (0.010) | -0.002 | (0.009) | -0.002 | (0.014) |
| 26.02-04.03 | 0.009 | (0.012) | -0.004 | (0.010) | 0.002 | (0.018) |
| 5-11.03 | Ref. | | Ref. | | Ref. | |
| 12-18.03 | 0.043^**^ | (0.020) | 0.049^***^ | (0.014) | 0.011 | (0.022) |
| 19-25.03 | 0.004 | (0.022) | -0.010 | (0.024) | 0.009 | (0.018) |
| 26.03-01.04 | -0.011 | (0.020) | -0.040 | (0.038) | 0.000 | (0.037) |
| N |  | 35,757 |  | 35,757 |  | 35,757 |
| R² |  | 0.3752 |  | 0.2557 |  | 0.2775 |

Note: The table reports estimates of the variable week of eq.1 (see section Methods). Country fixed effects are included. Clustered standard errors at the country level in are parentheses, * p < 0.1, ** p < 0.05, *** p < 0.01

**Table A.5: The effect of AstraZeneca temporary suspension: trust in Science**

| Trust in Science : full sample | (1)  Vaccine hesitancy: all | (2)  Vaccine hesitancy:  fear | (3)  Vaccine hesitancy: other reasons |
| --- | --- | --- | --- |
| Week 12-18.02 | -0.063^**^ (0.029) | -0.043^*^ (0.023) | -0.050^*^ (0.029) |
| Week 19-25.02 | -0.059^***^ (0.019) | -0.048^***^ (0.015) | -0.015 (0.025) |
| Week 26.02-04.03 | 0.018 (0.017) | 0.007 (0.012) | 0.007 (0.024) |
| Week 5-11.03 | Ref | Ref | Ref |
| Week 12-18.03 | 0.012 (0.020) | 0.013 (0.013) | 0.011 (0.022) |
| Week 19-25.03 | -0.040 (0.024) | -0.032^**^ (0.014) | -0.022 (0.019) |
| Week 26.03-01.04 | -0.029 (0.037) | -0.062^**^ (0.024) | 0.000 (0.039) |
|  |  |  |  |
| Low trust in science | 0.248^***^ (0.024) | 0.180^***^ (0.023) | 0.164^***^ (0.024) |
|  |  |  |  |
| 12-18.02 # low trust | -0.127^***^ (0.022) | -0.091^***^ (0.024) | -0.064^**^ (0.026) |
| 19-25.02 # low trust | 0.001 (0.017) | 0.012 (0.024) | -0.053^*^ (0.027) |
| 26.02-04.03 # low trust | -0.062^**^ (0.026) | -0.055 (0.034) | -0.036 (0.029) |
| 5-11.03 # low trust | Ref | Ref | Ref |
| 12-18.03 # low trust | 0.083^***^ (0.027) | 0.094^***^ (0.030) | 0.002 (0.032) |
| 19-25.03 # low trust | 0.070 (0.081) | 0.028 (0.058) | 0.052 (0.070) |
| 26.03-01.04 # low trust | 0.011 (0.050) | 0.026 (0.078) | -0.020 (0.070) |
|  |  |  |  |
| N | 35,757 | 35,757 | 35,757 |

Note: The table reports estimates as in eq.1 of the variable week interacted with the level of trust in science (see section Methods). Country fixed effects are included. Clustered standard errors at the country level in are parentheses, * p < 0.1, ** p < 0.05, *** p < 0.01

**Table A.6: The effect of AstraZeneca temporary suspension: heterogeneity analysis**

| Trust in Science: Gender | Female | | | Male | | |
| --- | --- | --- | --- | --- | --- | --- |
|  | (1)  all | (2)  fear | (3)  other | (4)  all | (5)  fear | (6)  other |
| Week 12-18.02 | -0.067^**^ (0.026) | -0.056^**^ (0.023) | -0.024 (0.020) | -0.059 (0.051) | -0.028 (0.042) | -0.079^*^ (0.044) |
| Week 19-25.02 | -0.055^***^ (0.014) | -0.046^***^ (0.012) | 0.011 (0.027) | -0.062 (0.036) | -0.050 (0.030) | -0.045 (0.029) |
| Week 26.02-04.03 | 0.014 (0.029) | -0.014 (0.025) | 0.039 (0.023) | 0.024 (0.037) | 0.031 (0.033) | -0.033 (0.030) |
| Week 5-11.03 | Ref | Ref | Ref | Ref | Ref | Ref |
| Week 12-18.03 | -0.026 (0.020) | 0.000 (0.026) | 0.001 (0.022) | 0.048^*^ (0.025) | 0.021 (0.019) | 0.020 (0.033) |
| Week 19-25.03 | -0.061^*^ (0.030) | -0.053^***^ (0.018) | -0.022 (0.018) | -0.025 (0.026) | -0.020 (0.021) | -0.029 (0.030) |
| Week 26.03-01.04 | -0.040 (0.029) | -0.092^***^ (0.029) | 0.012 (0.039) | -0.005 (0.060) | -0.007 (0.037) | -0.015 (0.049) |
|  |  |  |  |  |  |  |
| Low trust in science | 0.216^***^ (0.030) | 0.160^***^ (0.033) | 0.136^***^ (0.023) | 0.276^***^ (0.043) | 0.196^***^ (0.055) | 0.187^***^ (0.029) |
|  |  |  |  |  |  |  |
| 12-18.02 # low trust | -0.092^**^ (0.037) | -0.062 (0.042) | -0.056^**^ (0.024) | -0.153^**^ (0.056) | -0.110^*^ (0.063) | -0.066^*^ (0.035) |
| 19-25.02 # low trust | 0.037 (0.027) | 0.041 (0.034) | -0.053^*^ (0.030) | -0.036 (0.042) | -0.019 (0.037) | -0.046 (0.036) |
| 26.02-04.03 # low trust | -0.059 (0.046) | -0.026 (0.035) | -0.086^**^ (0.033) | -0.053 (0.033) | -0.082 (0.058) | 0.037 (0.036) |
| 5-11.03 # low trust | Ref | Ref | Ref | Ref | Ref | Ref |
| 12-18.03 # low trust | 0.118^***^ (0.032) | 0.137^***^ (0.036) | -0.007 (0.023) | 0.048 (0.031) | 0.052 (0.033) | 0.010 (0.068) |
| 19-25.03 # low trust | 0.109 (0.075) | 0.066 (0.064) | 0.090 (0.059) | 0.050 (0.102) | 0.015 (0.098) | 0.027 (0.093) |
| 26.03-01.04 # low trust | -0.001 (0.036) | -0.072 (0.108) | 0.041 (0.053) | 0.012 (0.069) | 0.109 (0.088) | -0.075 (0.096) |
|  |  |  |  |  |  |  |
| N | 22,455 | 22,455 | 22,455 | 13,302 | 13,302 | 13,302 |
|  |  | | |  | | |
| Trust in Science: Financial difficulties | No difficulties | | | Difficulties | | |
|  | all | fear | other | all | fear | other |
| Week 12-18.02 | -0.092^***^ (0.027) | -0.059^**^ (0.024) | -0.058^**^ (0.027) | -0.030 (0.035) | -0.027 (0.028) | -0.039 (0.037) |
| Week 19-25.02 | -0.103^***^ (0.022) | -0.066^***^ (0.017) | -0.051^**^ (0.019) | -0.003 (0.034) | -0.029 (0.028) | 0.034 (0.043) |
| Week 26.02-04.03 | -0.025 (0.018) | -0.026^**^ (0.010) | 0.003 (0.029) | 0.083^***^ (0.026) | 0.055 (0.033) | 0.015 (0.024) |
| Week 5-11.03 | Ref | Ref | Ref | Ref | Ref | Ref |
| Week 12-18.03 | -0.020 (0.022) | 0.009 (0.024) | -0.010 (0.017) | 0.048^*^ (0.026) | 0.013 (0.027) | 0.037 (0.030) |
| Week 19-25.03 | -0.066^**^ (0.032) | -0.065^**^ (0.027) | -0.036^**^ (0.016) | -0.019 (0.025) | 0.001 (0.022) | -0.007 (0.034) |
| Week 26.03-01.04 | -0.061 (0.040) | -0.069^***^ (0.020) | -0.027 (0.040) | 0.017 (0.060) | -0.053 (0.048) | 0.040 (0.054) |
|  |  |  |  |  |  |  |
| Low trust in science | 0.241^***^ (0.040) | 0.140^***^ (0.044) | 0.226^***^ (0.042) | 0.264^***^ (0.037) | 0.212^***^ (0.026) | 0.119^***^ (0.027) |
|  |  |  |  |  |  |  |
| 12-18.02 # low trust | -0.153^***^ (0.052) | -0.072 (0.047) | -0.150^**^ (0.056) | -0.101^***^ (0.031) | -0.092^***^ (0.022) | 0.007 (0.032) |
| 19-25.02 # low trust | 0.022 (0.040) | 0.039 (0.061) | -0.093^**^ (0.035) | -0.048 (0.053) | -0.019 (0.028) | -0.049 (0.070) |
| 26.02-04.03 # low trust | -0.005 (0.071) | 0.037 (0.062) | -0.101 (0.061) | -0.142^**^ (0.053) | -0.146^**^ (0.058) | 0.003 (0.034) |
| 5-11.03 # low trust | Ref | Ref | Ref | Ref | Ref | Ref |
| 12-18.03 # low trust | 0.113^**^ (0.049) | 0.152^***^ (0.047) | -0.054 (0.033) | 0.049 (0.043) | 0.059^**^ (0.027) | 0.031 (0.055) |
| 19-25.03 # low trust | 0.064 (0.093) | 0.051 (0.087) | 0.009 (0.104) | 0.090 (0.090) | 0.018 (0.094) | 0.094 (0.068) |
| 26.03-01.04 # low trust | 0.054 (0.051) | 0.147^**^ (0.071) | -0.088 (0.081) | -0.047 (0.111) | -0.073 (0.136) | 0.015 (0.094) |
|  |  |  |  |  |  |  |
| N | 20,074 | 20,074 | 20,074 | 15,683 | 15,683 | 15,683 |
|  |  |  |  |  |  |  |
| Trust in Science: Health | Healthy | | | Sick | | |
|  | all | fear | other | all | fear | other |
| Week 12-18.02 | -0.060^*^ (0.032) | -0.051^**^ (0.023) | -0.035 (0.028) | -0.054 (0.035) | -0.026 (0.030) | -0.049 (0.041) |
| Week 19-25.02 | -0.062^***^ (0.016) | -0.051^***^ (0.011) | -0.014 (0.019) | -0.041 (0.038) | -0.040 (0.027) | -0.004 (0.038) |
| Week 26.02-04.03 | -0.005 (0.029) | -0.024 (0.020) | 0.014 (0.018) | 0.060 (0.050) | 0.049 (0.050) | 0.005 (0.042) |
| Week 5-11.03 | Ref | Ref | Ref | Ref | Ref | Ref |
| Week 12-18.03 | 0.029 (0.021) | 0.011 (0.017) | 0.040^*^ (0.022) | -0.005 (0.030) | 0.021 (0.027) | -0.024 (0.030) |
| Week 19-25.03 | -0.005 (0.044) | -0.021 (0.017) | 0.008 (0.039) | -0.092^***^ (0.027) | -0.052^**^ (0.021) | -0.056^**^ (0.026) |
| Week 26.03-01.04 | -0.045 (0.040) | -0.080^***^ (0.029) | -0.005 (0.046) | -0.000 (0.072) | -0.021 (0.048) | -0.004 (0.065) |
|  |  |  |  |  |  |  |
| Low trust in science | 0.274^***^ (0.035) | 0.192^***^ (0.025) | 0.254^***^ (0.033) | 0.225^***^ (0.038) | 0.170^***^ (0.038) | 0.060 (0.043) |
|  |  |  |  |  |  |  |
| 12-18.02 # low trust | -0.133^***^ (0.039) | -0.088^**^ (0.038) | -0.138^***^ (0.038) | -0.139^***^ (0.034) | -0.108^***^ (0.030) | 0.001 (0.039) |
| 19-25.02 # low trust | -0.004 (0.039) | -0.008 (0.037) | -0.092^**^ (0.036) | -0.009 (0.053) | 0.025 (0.034) | -0.010 (0.052) |
| 26.02-04.03 # low trust | -0.027 (0.060) | -0.020 (0.047) | -0.100^**^ (0.040) | -0.119^**^ (0.055) | -0.103^*^ (0.051) | 0.022 (0.051) |
| 5-11.03 # low trust | Ref | Ref | Ref | Ref | Ref | Ref |
| 12-18.03 # low trust | 0.044 (0.046) | 0.050 (0.040) | -0.013 (0.050) | 0.131^**^ (0.048) | 0.144^**^ (0.062) | 0.022 (0.028) |
| 19-25.03 # low trust | 0.046 (0.105) | 0.050 (0.072) | -0.015 (0.094) | 0.109^*^ (0.060) | 0.005 (0.045) | 0.140^**^ (0.064) |
| 26.03-01.04 # low trust | 0.032 (0.042) | 0.054 (0.090) | -0.080 (0.084) | -0.072 (0.094) | -0.053 (0.077) | 0.020 (0.080) |
|  |  |  |  |  |  |  |
| N | 21,779 | 21,779 | 21,779 | 13,978 | 13,978 | 13,978 |
|  |  |  |  |  |  |  |
| Trust in Science: Urban/Rural | Rural | | | Urban | | |
|  | all | fear | other | all | fear | other |
| Week 12-18.02 | -0.044 (0.039) | -0.045 (0.031) | -0.044 (0.043) | -0.074^***^ (0.026) | -0.033^*^ (0.019) | -0.052^**^ (0.020) |
| Week 19-25.02 | -0.052^*^ (0.026) | -0.064^***^ (0.021) | 0.004 (0.035) | -0.057^**^ (0.022) | -0.027^*^ (0.015) | -0.027 (0.020) |
| Week 26.02-04.03 | 0.055^***^ (0.019) | 0.033^*^ (0.018) | 0.013 (0.038) | -0.020 (0.024) | -0.024^*^ (0.013) | 0.001 (0.019) |
| Week 5-11.03 | Ref | Ref | Ref | Ref | Ref | Ref |
| Week 12-18.03 | 0.030 (0.033) | 0.015 (0.020) | 0.017 (0.031) | -0.010 (0.025) | 0.012 (0.014) | 0.002 (0.022) |
| Week 19-25.03 | -0.036 (0.044) | -0.059^***^ (0.019) | -0.004 (0.041) | -0.041^**^ (0.020) | -0.006 (0.018) | -0.039^**^ (0.015) |
| Week 26.03-01.04 | -0.041 (0.062) | -0.072^*^ (0.041) | -0.022 (0.055) | -0.012 (0.032) | -0.043^**^ (0.021) | 0.022 (0.033) |
|  |  |  |  |  |  |  |
| Low trust in science | 0.260^***^ (0.024) | 0.166^***^ (0.041) | 0.201^***^ (0.039) | 0.241^***^ (0.042) | 0.206^***^ (0.034) | 0.120^***^ (0.022) |
|  |  |  |  |  |  |  |
| 12-18.02 # low trust | -0.149^***^ (0.026) | -0.070 (0.055) | -0.118^**^ (0.043) | -0.116^**^ (0.045) | -0.129^***^ (0.038) | -0.010 (0.033) |
| 19-25.02 # low trust | -0.007 (0.045) | 0.034 (0.043) | -0.100^*^ (0.054) | -0.009 (0.036) | -0.027 (0.038) | -0.010 (0.022) |
| 26.02-04.03 # low trust | -0.108^***^ (0.036) | -0.085 (0.069) | -0.067 (0.055) | -0.008 (0.034) | -0.024 (0.042) | 0.003 (0.020) |
| 5-11.03 # low trust | Ref | Ref | Ref | Ref | Ref | Ref |
| 12-18.03 # low trust | 0.069 (0.043) | 0.131^***^ (0.044) | -0.044 (0.029) | 0.102^**^ (0.045) | 0.044 (0.031) | 0.065 (0.068) |
| 19-25.03 # low trust | 0.088 (0.107) | 0.069 (0.061) | 0.037 (0.089) | 0.050 (0.060) | -0.016 (0.055) | 0.073 (0.087) |
| 26.03-01.04 # low trust | 0.054 (0.061) | 0.042 (0.124) | 0.004 (0.080) | -0.084 (0.093) | -0.023 (0.096) | -0.079 (0.069) |
|  |  |  |  |  |  |  |
| N | 12,368 | 12,368 | 12,368 | 23,389 | 23,389 | 23,389 |
|  |  |  |  |  |  |  |

| Trust in Science: Education | Low | | | High | | |
| --- | --- | --- | --- | --- | --- | --- |
|  | all | fear | other | all | fear | other |
| Week 12-18.02 | -0.056 (0.035) | -0.038 (0.028) | -0.049 (0.038) | -0.077^***^ (0.025) | -0.054^***^ (0.018) | -0.050^**^ (0.022) |
| Week 19-25.02 | -0.067^**^ (0.025) | -0.049^***^ (0.018) | -0.013 (0.036) | -0.052^***^ (0.018) | -0.053^***^ (0.019) | -0.027 (0.016) |
| Week 26.02-04.03 | 0.045^*^ (0.026) | 0.024 (0.023) | 0.017 (0.033) | -0.050^**^ (0.020) | -0.039^**^ (0.017) | -0.026^*^ (0.014) |
| Week 5-11.03 | Ref | Ref | Ref | Ref | Ref | Ref |
| Week 12-18.03 | 0.021 (0.028) | 0.016 (0.017) | 0.020 (0.031) | -0.019 (0.014) | 0.006 (0.011) | -0.017 (0.011) |
| Week 19-25.03 | -0.029 (0.022) | -0.021 (0.017) | -0.016 (0.017) | -0.057 (0.040) | -0.060^***^ (0.020) | -0.033 (0.036) |
| Week 26.03-01.04 | -0.087^**^ (0.034) | -0.104^***^ (0.030) | -0.035 (0.042) | 0.032 (0.039) | -0.022 (0.028) | 0.033 (0.038) |
|  |  |  |  |  |  |  |
| Low trust in science | 0.241^***^ (0.026) | 0.181^***^ (0.030) | 0.160^***^ (0.028) | 0.285^***^ (0.029) | 0.183^***^ (0.039) | 0.187^***^ (0.039) |
|  |  |  |  |  |  |  |
| 12-18.02 # low trust | -0.119^***^ (0.029) | -0.092^**^ (0.038) | -0.062^*^ (0.030) | -0.172^***^ (0.037) | -0.093^*^ (0.053) | -0.091^*^ (0.052) |
| 19-25.02 # low trust | 0.035^*^ (0.019) | 0.026 (0.025) | -0.045 (0.040) | -0.126^***^ (0.030) | -0.038 (0.033) | -0.098^***^ (0.034) |
| 26.02-04.03 # low trust | -0.074^**^ (0.028) | -0.067 (0.044) | -0.037 (0.033) | -0.069^**^ (0.029) | -0.038 (0.041) | -0.063^**^ (0.025) |
| 5-11.03 # low trust | Ref | Ref | Ref | Ref | Ref | Ref |
| 12-18.03 # low trust | 0.096^***^ (0.033) | 0.120^***^ (0.031) | 0.008 (0.036) | 0.000 (0.039) | -0.022 (0.053) | -0.060 (0.046) |
| 19-25.03 # low trust | 0.083 (0.094) | 0.024 (0.075) | 0.069 (0.072) | -0.012 (0.047) | 0.025 (0.054) | -0.047 (0.058) |
| 26.03-01.04 # low trust | 0.098^*^ (0.057) | 0.054 (0.105) | 0.053 (0.083) | -0.156^*^ (0.088) | -0.009 (0.064) | -0.161^**^ (0.059) |

| N | 12,368 | 12,368 | 12,368 | 23,389 | 23,389 | 23,389 |
| --- | --- | --- | --- | --- | --- | --- |

Note: The table reports estimates as in eq.1 of the variable week interacted with the level of trust in science (see section Methods). Separate estimations are displayed for each characteristic: gender, financial difficulties, health status, urban/rural, and educational attainment. Country fixed effects are included. Clustered standard errors at the country level in are parentheses, * p < 0.1, ** p < 0.05, *** p < 0.01

**Table A.7: Descriptive statistics**

| **Variable** | **Mean** | **Sd.** | **Min** | **Max** | **Variable** | **Mean** | **sd.** | **Min** | **Max** |
| --- | --- | --- | --- | --- | --- | --- | --- | --- | --- |
| Vaccine hesitancy: all | 0.255 | 0.4358 | 0 | 1 | Region: The open countryside | 0.115 | 0.3191 | 0 | 1 |
| Vaccine hesitancy: fear | 0.184 | 0.3873 | 0 | 1 | Region: A village/small town | 0.414 | 0.4926 | 0 | 1 |
| *Vaccination has negative impact on health* | 0.073 | 0.2604 | 0 | 1 | Region: A medium to large town | 0.205 | 0.4036 | 0 | 1 |
| *No trust in safety of vaccination* | 0.159 | 0.3657 | 0 | 1 | Region: A city or city suburb | 0.266 | 0.4419 | 0 | 1 |
| Vaccine hesitancy: other | 0.146 | 0.3529 | 0 | 1 | Income sufficient : With great difficulty | 0.116 | 0.3201 | 0 | 1 |
| *Risk of COVID is exaggerated* | 0.113 | 0.3171 | 0 | 1 | Income sufficient: With difficulty | 0.113 | 0.3161 | 0 | 1 |
| *COVID does not exist* | 0.019 | 0.1363 | 0 | 1 | Income sufficient: With some difficulty | 0.212 | 0.4087 | 0 | 1 |
| *Other reason* | 0.033 | 0.1791 | 0 | 1 | Income sufficient: Fairly easily | 0.235 | 0.4241 | 0 | 1 |
| Trust in news | 4.246 | 2.4866 | 1 | 10 | Income sufficient: Easily | 0.192 | 0.3939 | 0 | 1 |
| Trust in police | 5.748 | 2.7256 | 1 | 10 | Income sufficient: Very easily | 0.132 | 0.3390 | 0 | 1 |
| Trust in government | 3.974 | 2.8698 | 1 | 10 | Employment status: Employee | 0.436 | 0.4959 | 0 | 1 |
| Trust in EU | 4.692 | 2.8427 | 1 | 10 | Employment status: Self-employed | 0.067 | 0.2507 | 0 | 1 |
| Trust in Health system | 5.963 | 2.6706 | 1 | 10 | Employment status: Unemployed | 0.105 | 0.3066 | 0 | 1 |
| Trust in social media | 3.389 | 1.9875 | 1 | 10 | Employment status: Retired | 0.260 | 0.4385 | 0 | 1 |
| Trust in Science | 7.274 | 2.4575 | 1 | 10 | Employment status: Other | 0.132 | 0.3385 | 0 | 1 |
| Trust in pharmaceutical firms | 4.660 | 2.6438 | 1 | 10 | Education: Primary | 0.052 | 0.2221 | 0 | 1 |
| Trust in people | 5.020 | 2.5379 | 1 | 10 | Education: Secondary | 0.669 | 0.4705 | 0 | 1 |
| Main source of information: Press | 0.343 | 0.4748 | 0 | 1 | Education: Tertiary | 0.279 | 0.4483 | 0 | 1 |
| Main source of information: Radio | 0.069 | 0.2539 | 0 | 1 | COVID-19: tested positive | 0.081 | 0.2734 | 0 | 1 |
| Main source of information: Social media | 0.166 | 0.3723 | 0 | 1 | COVID-19: someone close tested positive | 0.400 | 0.4899 | 0 | 1 |
| Main source of information: Other media | 0.114 | 0.3172 | 0 | 1 | COVID-19: someone close died | 0.105 | 0.3072 | 0 | 1 |
| Female | 0.521 | 0.4996 | 0 | 1 | COVID-19: someone close died from another cause | 0.299 | 0.4576 | 0 | 1 |
| Age: 18-29 | 0.149 | 0.3559 | 0 | 1 | Health: Very good | 0.141 | 0.3476 | 0 | 1 |
| Age: 30-49 | 0.327 | 0.4693 | 0 | 1 | Health: Good | 0.459 | 0.4983 | 0 | 1 |
| Age: 50-64 | 0.281 | 0.4497 | 0 | 1 | Health: Fair | 0.309 | 0.4621 | 0 | 1 |
| Age: 65+ | 0.242 | 0.4285 | 0 | 1 | Health: Bad | 0.078 | 0.2686 | 0 | 1 |
|  |  |  |  |  | Health: Very bad | 0.013 | 0.1123 | 0 | 1 |
| N | 35,757 |  |  |  |  | 35,757 |  |  |  |
| N weighted | 36145.76 |  |  |  |  | 36145.76 |  |  |  |

Note: Weighted descriptive statistics of the variables of interested. Survey weights are used based on age, gender, education, and urbanization levels, to obtain representative data of the demographic profile of the European Union as a whole and of the member states Sd=Standard deviation.
